# Cooperative ligand binding in a bacterial heme-based oxygen sensor

**DOI:** 10.1016/j.jbc.2025.111025

**Published:** 2025-12-08

**Authors:** Nushrat J. Hoque, Sarah R. Pope, Varun Venkatakrishnan, David O. Olori, Noah A. Brady, Dayna C. Patterson, Ganesh S. Anand, Yilin Liu, Amie K. Boal, Emily E. Weinert

**Affiliations:** 1 Department of Chemistry, Pennsylvania State University, University Park, Pennsylvania, USA; 2 Department of Biochemistry and Molecular Biology, Pennsylvania State University, University Park, Pennsylvania, USA; 3 Department of Chemistry, University of Akron, Akron, Ohio, USA

**Keywords:** cooperative ligand binding, bacterial sensor globin, heme, HDX-MS, resonance Raman

## Abstract

Bacteria modulate essential phenotypes in response to external signals such as the availability of molecular oxygen (O_2_). A class of direct O_2_-sensing heme proteins, globin-coupled sensors, have been implicated in O_2_-dependent regulation of pathogenic phenotypes, including biofilm formation, motility, and virulence. While cooperative O_2_ binding is well known in both mammalian and prokaryotic hemoglobins, cooperative ligand binding previously has not been observed in bacterial sensor globins. This study explores the O_2_-dependent allosteric communication between globin domains in the globin-coupled sensor protein from *Pectobacterium carotovorum* (*Pcc*GCS) through equilibrium O_2_-binding measurements, X-ray crystallography, resonance Raman spectroscopy, and hydrogen-deuterium exchange mass spectrometry. Based on these experiments, we propose a model of allosteric regulation of O_2_ binding that is directed by subtle changes in distal heme pocket protein conformation and transduced through dynamics of helices at the dimer interface of the *Pcc*GCS sensor globin. Together, this work identifies cooperative ligand binding in a family of bacterial heme proteins, which could allow the bacteria to more robustly respond to small changes in O_2_ levels. Furthermore, this work highlights the importance of heme pocket residues in transducing the O_2_ binding event within the dimer and suggests a pathway for signal transduction in dimeric myoglobin-like sensor proteins.

Allostery in signaling proteins enables environmental or cellular signals to modulate catalysis, oligomerization, and ligand binding. In multi-domain proteins, alterations in one domain can induce allosteric changes in another domain. This phenomenon allows minor modifications, such as ligand binding or post-translational modifications, to regulate biochemical processes over considerable distances within the protein structure (1, 2). In addition, proteins with multiple binding sites for the same ligand have the potential to exhibit cooperative allosteric regulation. Cooperativity is a specific type of allostery wherein binding of a ligand to a biomolecule alters the binding affinity of an additional ligand of the same identity at another binding site (3, 4). Positive cooperativity has been extensively studied in mammalian hemoglobins and allows for increased oxygen (O_2_) affinity upon binding of an initial O_2_ molecule to one of the four heme sites (5, 6). The mechanism of hemoglobin cooperativity has been modeled through either a two-state, concerted mechanism (Monod-Wyman-Changeux model (7)) or a sequential, induced fit mechanism (Pauling-Koshland-Némethy-Filmer model (8)), which attempts to account for the conformational heterogeneity of the macromolecules upon oxygenation. While some bacterial hemoglobins have been demonstrated to bind O_2_ cooperatively (9, 10), the full dynamics of hemoglobin allostery remains unclear (11). Furthermore, positive cooperativity has not been reported for other members of the globin superfamily in prokaryotes.

Within bacteria, a family of multi-domain, heme-based signaling proteins termed globin-coupled sensors (GCSs) exhibit allosteric regulation of catalytic activity (12, 13). GCS proteins consist of a sensor globin domain connected to one or more output domains, such as kinases, methyl-accepting chemotaxis protein (MCP; involved in motility), or diguanylate cyclase (DGC; synthesizes bacterial second messenger and biofilm regulator, bis-(3′-5′)-cyclic dimeric guanosine monophosphate) (14, 15, 16). Changes to the ligation and/or redox state of the sensor globin domain heme alter the activity of the output domain (14, 17). For example, coupling an O_2_ (or NO/CO/redox) sensing domain to an output domain allows the bacteria to identify changes in their gaseous environment and respond to maximize survival. Ligand-dependent catalytic activity is likely driven by conformational rearrangements propagated throughout the domains of oligomeric GCS proteins (18, 19, 20).

No high-resolution structures of full-length GCS protein have been published to date; lower resolution techniques have demonstrated that GCS proteins form multimers in solution (12, 21) and that the formation of multimers impacts catalytic activity (14). Additionally, X-ray crystallographic analysis of isolated GCS globin domains demonstrated the formation of dimers *in crystallo* (22, 23, 24, 25). While homo-dimeric hemoglobins from bacteria that exhibit cooperative O_2_ binding have been identified (9, 10), bacterial sensor globins are more structurally similar to mammalian monomeric myoglobins (14), which do not exhibit cooperativity. Dimeric bacterial sensor globins, therefore, allow us to address the question of whether oligomerization of myoglobin-like proteins can result in allosteric communication between monomers to regulate heme ligand binding, as well as to identify structural features involved in the inter-heme communication.

The multimeric GCS proteins from *Pectobacterium carotovorum* (*Pcc*GCS, DGC output domain) and *Bordetella pertussis* (*Bpe*GReg, DGC output domain) exhibit biphasic O_2_ dissociation rates (21), supporting the possibility of allosteric communication of heme ligand binding between the sensor globin domains. The non-equivalency of O_2_ dissociation from the hemes has been attributed to differences in hydrogen bonding interactions within the heme pockets, potentially due to allosteric regulation through the globin dimer interface (21, 26). Previous studies on the GCS from *Bacillus subtilis*, HemAT-*Bs* (MCP output domain), also identified two rates for O_2_ dissociation from the heme, suggesting that the hemes are non-equivalent (12). Equilibrium binding studies yielded biphasic binding curves; however, the measured Hill coefficient for HemAT-*Bs* was less than one (n = 0.45), corresponding to either independent binding to subunits with different O_2_ affinities or negative cooperativity (12).

To date, O_2_-binding cooperativity has not been examined in other GCS proteins. Therefore, to investigate possible allosteric regulation of heme ligand binding in *Pcc*GCS and the isolated globin domain, *Pcc*Globin (Fig. 1), this study employs a combination of equilibrium ligand and redox titrations, amide hydrogen-deuterium exchange mass spectrometry (HDX-MS), resonance Raman (rR) spectroscopy, and X-ray crystallography. The results demonstrate that the dimeric sensor globin *Pcc*GCS exhibits cooperative O_2_ and cyanide (CN^-^) binding, highlight residues and rearrangements required for allosteric communication between hemes and suggest a mechanism for communication between hemes in sensor globins. These findings provide new information about allostery in heme proteins, address open questions regarding cooperativity in myoglobin-like fold, and yield insights into the mechanism by which ligation/redox state of the sensor globin heme is propagated through full-length GCS proteins to control bacterial behaviors.

**Figure 1:**
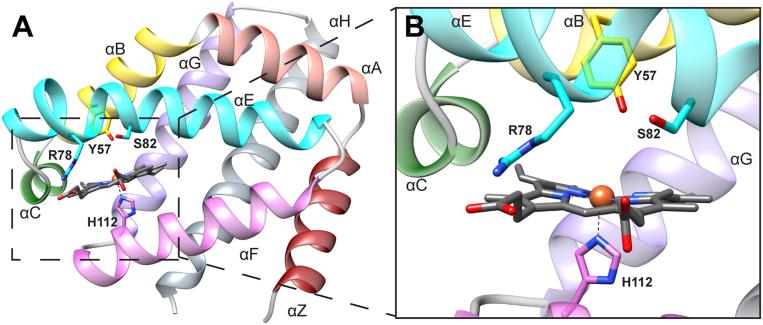
**Homology Model of *Pcc*GCS globin domain monomer**. N-terminal helices *A* (*orange*) and Z (*red*) are predicted to be flexible. The heme pocket is composed of helices *B* (*yellow*), C (*green*), E (*cyan*), and F (*pink*). Helices G (*purple*) and H (*gray*) on the C-terminal form the dimer interface. The proximal histidine, H112, is conserved in GCS proteins. The distal tyrosine, Y57, and serine, S82, residues have been implicated in ligand binding on the distal face (Fig. S1). The homology model was made for *Pcc*Globin using *Bpe*Globin crystal structure (6M9A) (25) and the Protein Homology/analogY Recognition Engine V2.0 (PHYRE2) tool (63).

## Results

### *Pcc*GCS cooperatively binds O_2_ and CN^-^ ligands

*Pcc*GCS forms oligomers in solution, allowing for potential communication between heme moieties. *Pcc*GCS forms primarily tetramers with smaller percentages of dimers and octamers, while *Pcc*Globin (the isolated sensor globin domain of *Pcc*GCS) is dimeric (26). To investigate possible ligand binding allostery within the globin domains in *Pcc*GCS and *Pcc*Globin, equilibrium O_2_ titrations were performed, and the data were fit to a two-site model and the Hill equation (27). The overall *K*_d_ of full-length *Pcc*GCS was 1.28 *μ* M with a Hill coefficient (n) of 1.39, demonstrating positive cooperativity (Table 1, Fig. S1, Table S1). The equilibrium binding affinity of the isolated globin domain was then measured to determine if the globin dimer interface was sufficient for cooperativity or if contacts with the middle and DGC domain contacts were required. *Pcc*Globin also exhibited cooperative O_2_ binding, with minor changes in affinity (*K*_d_ = 1.16 μM) and Hill coefficient (n = 1.51) (Table 1). The high n value and biphasic character (Fig. S1) indicate that O_2_ binding occurs through a positively cooperative mechanism. Therefore, the middle and cyclase domain contacts are not required to transduce cooperativity between the globin domains.

**Table 1:** Equilibrium oxygen affinity of *Pcc*GCS WT and variants determined through direct titrations and fit to the Hill equation

Protein	*K*_d_ (μM)	n (Hill coefficient)
sperm whale myoglobin (swMb) (25)	0.83–1.5	0.95 ± 0.05
HemAT-*Bs* (12)	10	0.45
*Pcc*GCS	1.28 ± 0.03	1.39 ± 0.04
*Pcc*Globin	1.16 ± 0.04	1.51 ± 0.05
*Pcc*Globin S82 A	0.35 ± 0.02	0.70 ± 0.03
*Pcc*GCS S82 A	0.28 ± 0.02	0.48 ± 0.02

Values are reported as the average of triplicates ± S.D.

In the distal heme pocket, tyrosine and serine residues have been implicated in ligand stability and recognition in several GCS proteins, including *Pcc*GCS (25, 26), *Bpe*GReg (28), and HemAT-*Bs* (29) (Figs. 1, S2). Therefore, mutations to the distal pocket were used to probe the influence of hydrogen bonding residues on O_2_ binding cooperativity. While *Pcc*GCS and *Pcc*Globin exhibit positive cooperativity for O_2_ binding, mutations to the distal serine residue (S82A) in both the full-length and the isolated globin domain led to a loss of cooperativity. The *Pcc*GCS S82A variant yielded a *K*_d_ of 0.28 mM and Hill coefficient of 0.48, while the *Pcc*Globin S82 A variant resulted in a *K*_d_ of 0.35 mM and Hill coefficient of 0.70 (Table 1). Distinguishing between negative cooperativity and two independent sites is challenging; however, the decrease in Hill coefficients below one for the full-length GCS and isolated globin domain demonstrates that the S82 residue is essential for cooperative O_2_ binding.

Mutation of the distal pocket tyrosine (Y57) results in loss of stable O_2_ binding and rapid heme auto-oxidation (26), prohibiting equilibrium O_2_ binding measurements for the Y57F variant. Instead, the effect of the conserved distal tyrosine was probed using equilibrium CN^-^ titrations to Fe(III) *Pcc*Globin. CN^-^ has been widely used as a molecular mimic for O_2_ binding to P450s (30, 31, 32) due to the stability of the Fe(III)-CN complex and the similar binding angle. The Fe(II)-O_2_ complex in globins, as measured by resonance Raman spectroscopy, is very similar to that of P450s, demonstrating ferric-superoxide-like character (33, 34, 35). Finally, CN^-^ has been shown to bind ferric heme at a slightly bent angle, as observed in the crystal structures of Fe(III)-CN states of sensor globins from HemAT-*Bs* and *Af*GcHK, the globin-coupled histidine kinase from *Anaeromyxobacter* sp. Fw109-5 (23, 24, 36). Wild-type *Pcc*Globin exhibited positive cooperativity for CN^-^ binding, with a *K*_d_ of 0.79 μM and a Hill coefficient of 1.44. The CN^-^ ligand binding to the *Pcc*Globin Y57F variant remained cooperative (n = 1.53) but was considerably weaker than WT (*K*_d_ = 42 μM), underscoring the importance of the Y57 hydrogen bonding capability in stabilizing heme ligand binding. The S82A variant did not affect CN^-^ affinity and, similar to the Y57F variant, did not impact the Hill coefficient (*K*_d_ = 0.78 μM; n = 1.41) (Table 2).

**Table 2:** Equilibrium cyanide affinity of *Pcc*GCS WT and variants determined through direct titrations and fit to the Hill equation

Protein	K_d_ (μM)	n (Hill coefficient)
*Pcc*Globin WT	0.79 ± 0.03	1.44 ± 0.05
*Pcc*Globin Y57F	42 ± 6	1.53 ± 0.06
*Pcc*Globin S82A	0.78 ± 0.03	1.41 ± 0.04

Values indicate an average of triplicates ± S.D.

As conformational shifts can be tied to changes in the redox potential of the heme iron (37, 38), the potential for redox cooperativity between the two hemes in a dimer was also investigated. In redox cooperativity, changes in the protein conformation upon ligand binding alter the midpoint potential for the oxidation of the second site. Oxidative and reductive titrations of *Pcc*GCS previously showed midpoint potentials of −7 mV and 246 mV vs. SHE (Figs. S3, S4) (39). The first potential, −7 mV vs SHE, falls within the range of other histidyl-ligated heme proteins (20), while the high second midpoint potential suggests that the heme environments are non-equivalent. The asymmetry of the Nernst plot from redox titrations (Fig. S5, black line) supports a multi-state model, as seen in hemoglobin (40). No redox cooperativity was detected (n_max_=1.14 ± 0.30, Table S2) in the WT protein, but the complexity of the spectra made it challenging to resolve the redox transitions through spectroelectrochemical titrations. Given the differences in the UV-Vis spectra of the full length Y57F variant *versus* globin variant but the similar midpoint potentials of the lower transition (Figs. S3, S4), it is likely Y57 plays an important role in the change in redox state. Based on the loss of the higher potential midpoint in the Y57F variant, it is likely that either the distal tyrosine either directly interacts with the heme iron or potentiates heme ligation by another residue during the redox titrations, resulting in a different midpoint potential. The small fraction of protein that exhibits the higher midpoint potential may be due to changes in protein conformation/dynamics due to direct interaction with the electrode. The heme pocket conformational dynamics make it challenging to directly assess redox cooperativity, suggesting that while *Pcc*GCS exhibits cooperative O_2_/CN^-^ binding, the non-equivalence in the heme redox potentials is not tied to the allosteric regulation of ligand binding.

### Heme pocket interactions involved in cooperative ligand binding

**Figure 2:**
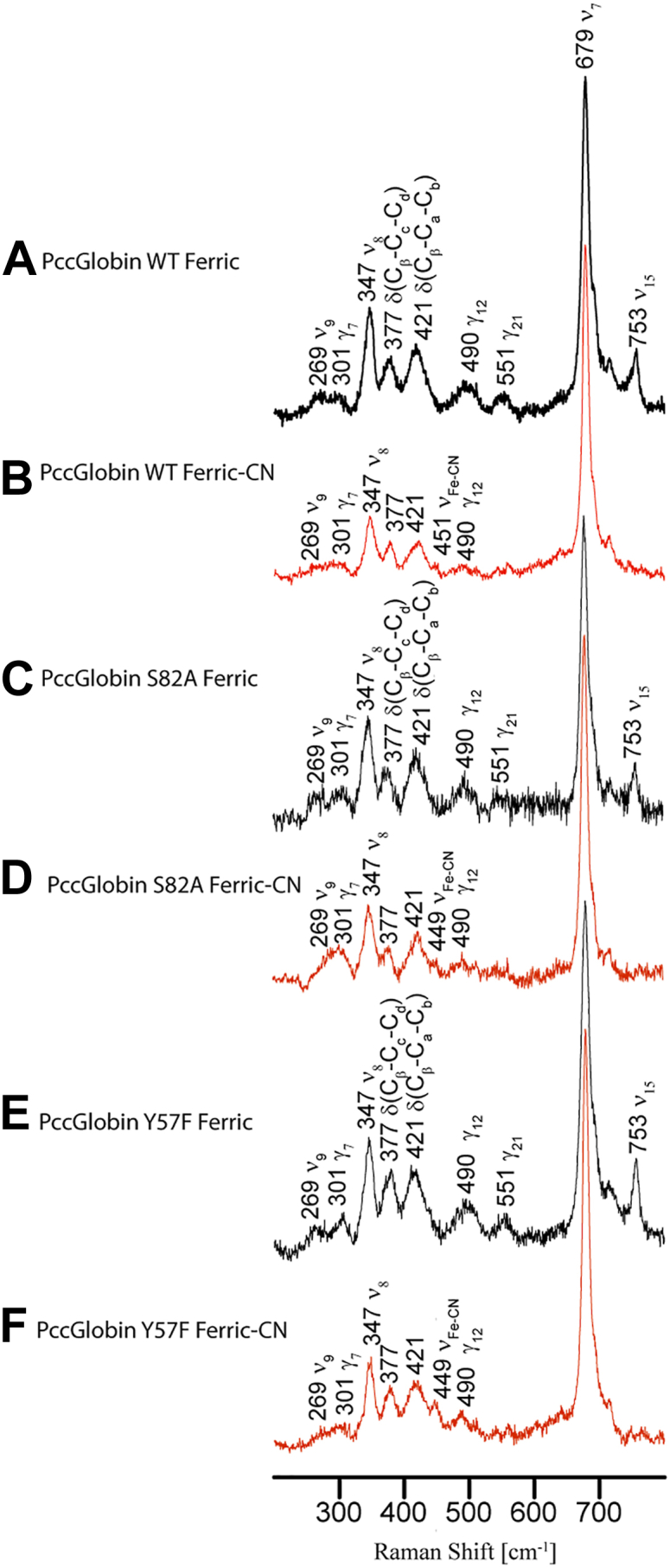
**rR *Pcc*GCS and *Pcc*Globin Variants in Fe(III)-H_2_O and Fe(III) CN states**. *A*, *Pcc*Globin WT Fe(III) (*B*) *Pcc*Globin WT Fe(III)-CN (*C*) *Pcc*Globin S82A Fe(III) (*D*) *Pcc*Globin S82A Fe(III)-CN (*E*) *Pcc*Globin Y57F Fe(III) (*F*) *Pcc*Globin Y57F Fe(III)-CN.

We hypothesized, based on the cooperative O_2_ vs CN^-^ binding results, that mutation of distal pocket hydrogen bond donors disrupted interactions with bound O_2_ but did not affect CN^-^ due to the differences in ligand electrostatics. Therefore, resonance Raman (rR) spectroscopy was used to investigate potential differences in heme pocket interactions and conformation upon O_2_ vs. CN^-^ ligand binding and to probe the effects of distal pocket mutations in different ligation/oxidation states. rR experiments were performed on *Pcc*Globin, *Pcc*Globin S82A, and *Pcc*Globin Y57F in both Fe(III) and Fe(III)-CN ligated states. The rR spectra of WT *Pcc*Globin, S82A, and Y57F Fe(III) species are shown in Figure 2, *A*, *C*, and *E*, respectively. In the WT Fe(III) protein, the heme skeletal modes are assigned to ν_7_ (679 cm^−1^), ν_8_ (347 cm^−1^), and ν_15_ (753 cm^−1^). The band observed at 377 cm^−1^ is attributed to the heme propionate bending coordinates, while the band appearing at 421 cm^−1^ is typically assigned to the “vinyl bending” mode. Several significant enhanced out-of-plane (OOP) modes are seen at 301, ∼490, and 551 cm^−1^, which are assigned to the γ_7_, γ_12_, and γ_21_ modes, respectively. Specifically, the γ_7_ mode, which belongs to A_2u_ symmetry, is indicative of a 'pyrrole tilt' heme out-of-plane mode, while the strong γ_21_ mode suggests the presence of a waving distortion of the heme macrocycle. These modes clearly indicate non-planar deformations of the heme, which are expected to result in distinct chemical and physical properties of the proteins (41, 42). Interestingly, mutating these key active site residues has minimal effects on either the heme modes or the planarity of the heme macrocycle, as reflected by the similar spectral patterns. However, some minor differences were seen in the relatively intensity of the propionate mode (377 cm^-1^), which is slightly increased in the Y57F mutants (Fig. 2*E*), suggesting a stronger H-bond interaction with a nearby amino acid residue, likely R78, based on previous studies (19, 25).

**Figure 3:**
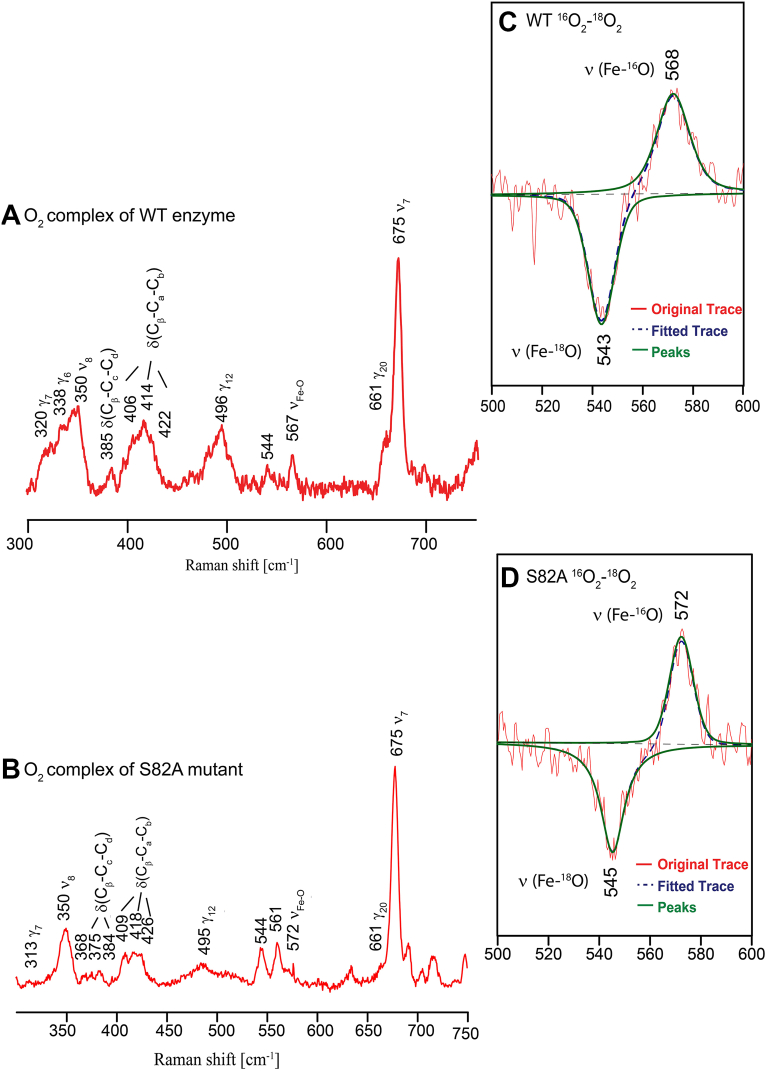
**rR spectra of Fe(II)-O_2_*Pcc*GCS.** The rR spectra of Fe(II)-O_2_ WT (*A*) and S82A mutant (*B*). Spectra were obtained using 413 nm excitation line. The inset shows the ^16^O_2_-^18^O_2_ difference traces of WT (*C*) and S82A *Pcc*Globin (*D*), respectively. The [^16^O_2_-^18^O_2_] difference trace was generated by subtracting the two absolute spectral traces, where all non-shifted features (*i*.*e*., those not associated with the Fe-O-O fragment) effectively cancel, and only the targeted ν(Fe-O) modes are observed, allowing for observation of accurate frequencies for the Fe-O stretch. Spectra were deconvoluted with Grams 32/AI software using a peak fitting procedure, employing 50/50% Gaussian/Lorentzian functions. *Red solid line*, experimental data; *blue dotted line*, fitted spectra; *green solid line*, modes associated with ν(Fe-O) modes.

Given the differences in cooperative O_2_ binding, rR spectroscopy was used to further probe the effects of heme pocket mutations on *Pcc*Globin WT and S82A heme conformation in the Fe(II)-O_2_ state. Based on the rR spectral traces for the Fe(II)-O_2_ complexes (Fig. 3, *A* and *B*), the S82A mutation causes substantial decreases in the number and intensities of the heme oop modes (γ_6_, γ_7_, γ_12_ and γ_20_), as compared to the WT protein, indicating a flattening of the heme, that may be caused by changes to heme pocket hydrogen bonding networks and conformation (19, 22, 43).

To examine the perturbations of the hydrogen bonding network upon O_2_ binding, which we hypothesized could cause the loss of cooperativity in the S82A mutant, the Fe-O_2_ stretching modes were probed in *Pcc*GCS WT and the globin domain variants (25, 26). As shown for Fe(II)-O_2_ WT *Pcc*GCS (Fig. 3*C*), the Fe-O_2_ stretching modes (ν(Fe-^16^O) = 568 cm^-1^; ν(Fe-^18^O) = 543 cm^-1^) are consistent with a hydrogen-bonded Fe-O-O conformer. In contrast, the rR ^16^O_2_–^18^O_2_ difference spectra obtained for Fe(II)-O_2_ S82A *Pcc*Globin (Fig. 3*D*) exhibit a ν(Fe-O) mode (ν(Fe-^16^O) = 572 cm^-1^; ν(Fe-^18^O) = 545 cm^-1^) that is shifted up by 4 cm^-1^, as compared WT (29).

Given that rR spectra only showed changes within the heme pocket for *Pcc*GCS S82A Fe(II)-O_2_, but not Fe(III) and Fe(III)-CN ligation states, X-ray crystallography was used to determine if the mutation caused conformational or structural changes outside of the heme pocket and if the Fe(II)-O_2_ vs. Fe(III)-CN ligation states resulted in structural differences. Unfortunately, *Pcc*Globin has been recalcitrant to crystallization; therefore, a homologous sensor globin (*Bpe*Globin) from the *B*. *pertussis* GCS (*Bpe*GReg) was used for structural studies because it shares key heme pocket hydrogen bonding and heme-interacting residues (Fig. 1*B*, S1; 31% overall sequence identity to the *Pcc*GCS globin domain) and has previously been found to readily crystallize. Additionally, *Bpe*GReg shares many similarities with *Pcc*GCS, including the domain organization, multimer formation in solution (25), and biphasic O_2_ dissociation (21), suggesting the presence of non-equivalent heme environments and the possibility of cooperative ligand binding. As homology models of *Pcc*Globin based on each crystallized sensor globin yield only minor differences, *Bpe*Globin likely provides a reasonable structural model for *Pcc*Globin. However, differences in amino acid identity could potentially cause unforeseen structural differences, supporting the need for biochemical experiments on *Pcc*Globin.

**Figure 4:**
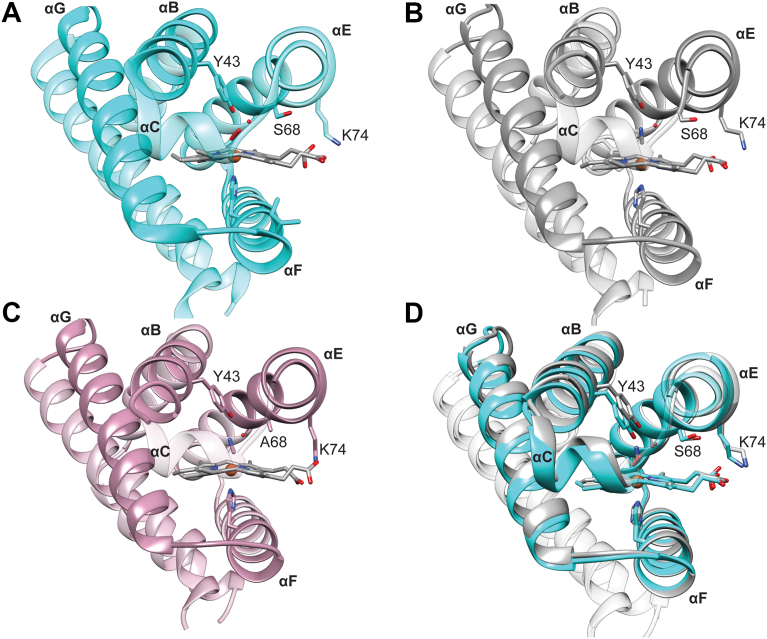
**Crystal Structures of *Bpe*Globin WT and S68A Fe(III)-CN**. *A*, *Bpe*Globin WT Fe(II)-O_2_ (*cyan*) structure from PDB 6m9a with labeled helices. *B*, *Bpe*Globin WT Fe(III)-CN (*gray*; PDB: 9DSE) structure from this work. *C*, *Bpe*Globin S68A Fe(III)-CN (*pink*; PDB: 9DSF) structure from this work. *D*, overlay of chain *A*, *Bpe*Globin WT Fe(III)-CN (*gray*) with chain *A*, *Bpe*Globin WT Fe(II)-O_2_ (*cyan*) (PDB 6m9a). Structures are aligned on the proximal helix, helix F using ChimeraX. Shifts in the helixes can be visualized in helices *B*, *C*, *E*, and *G*.

*Bpe*Globin WT and the serine variant (S68A) were crystallized in the Fe(III)-CN ligation state. Both proteins crystallized as dimers in the C_121_ space group, with 2.08 Å and 1.85 Å resolution, respectively (Figs. 4, S6, S7; Table S3). As previously observed in the structures *Bpe*Globin WT Fe(II)-O_2_ and Fe(III)-H_2_O (PDB 6m9a), the overall sensor globin structure was maintained for WT and S68A Fe(III)-CN structures (Fig. 4). When comparing the structures *Bpe*Globin WT in the three ligation states, the *Bpe*Globin Fe(III)-CN structures only exhibited a small shift in the distal helices relative to the helix containing the proximal histidine, as compared to Fe(II)-O_2_ and Fe(III)-H_2_O structures, due to the linear binding of the CN^-^ ligand, as compared to bent binding mode for O_2_ (Figs. 4*D*, S7, S8; Tables S4, S5; iron-ligand bond angles: WT Fe(III)-CN = 160.5°, S68 A Fe(III)-CN = 164.6°, WT Fe(II)-O_2_ (chain A) = 122.0°, WT Fe(II)-O_2_ (chain B) = 138.1°). Comparison of the *Bpe*Globin WT (Fig. 4*B*) and Fe(III)-CN S68A (Figs. 4*C*, S9) structures yielded only minor, non-significant changes in hydrogen bonding distances between the distal tyrosine and CN^-^ ligand ordered distal water (19). The structural data support the rR studies and confirm that mutation of the serine residue does not strongly alter the hydrogen bonding network or conformation of Fe(III)-CN *Bpe*Globin.

### Ligand binding causes small but widespread changes in sensor globin flexibility

To gain further insight into the interactions allowing for ligand binding cooperativity and differences between ligation states, amide hydrogen-deuterium exchange mass spectrometry (HDX-MS) was used to compare the conformational changes of *Pcc*Globin in the Fe(III)-H_2_O, Fe(III)-CN, Fe(II)-O_2_ and Fe(II)-unligated states. HDX-MS analysis yielded 45 peptides, with a total sequence overage of 82.7% coverage and 2.89 redundancy value, allowing for high significance in differences (Fig. S10). When comparing any two ligation/oxidation states, small differences in uptake were observed across the globin domain, suggesting that ligand binding and allosteric communication between hemes involve a network of amino acid interactions.

Compared to the three other ligation and oxidation states, the Fe(III)-H_2_O state exhibited the largest increase in deuterium uptake in residues 60 to 68, suggesting increased flexibility of the distal pocket (Fig. S11). In contrast, cyanide binding (Fe(III)-CN state) resulted in decreased deuterium exchange in the distal pocket (composed of helices B, C, and E, with Y57 situated on helix B, and S82 on helix E; 1.41 Da at t = 3 min; Table S6), relative to Fe(III)-H_2_O, which represents reduced conformational dynamics within the distal heme pocket. Additional low magnitude decreases in deuterium exchange were observed in the proximal helix, demonstrating that ligand binding in the ferric state results in overall rigidification of the *Pcc*Globin scaffold (Fig. S11).

**Figure 5:**
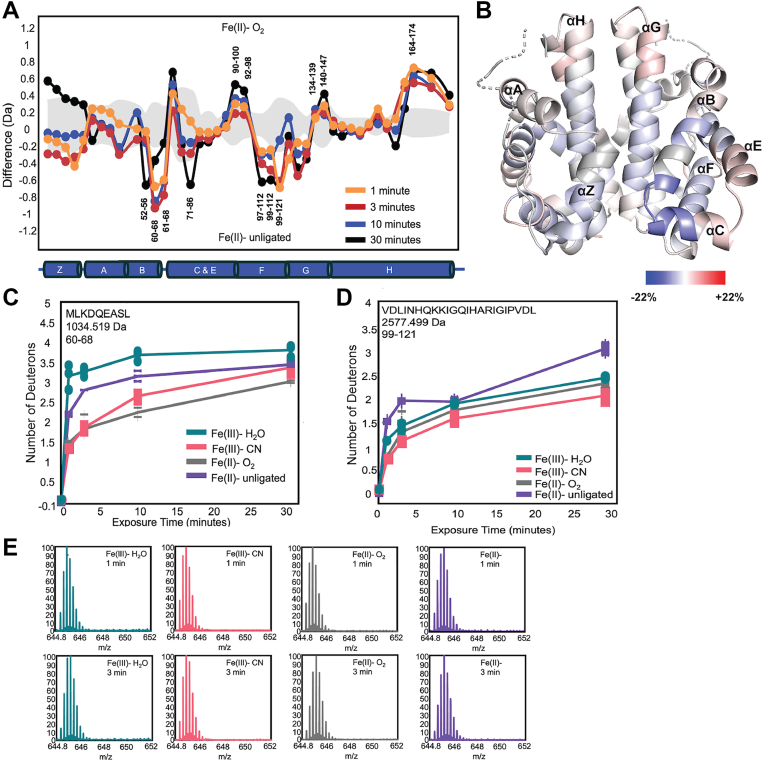
**HDX-MS Comparison of Fe(II)-O_2_ vs. Fe(II)-unligated PccGlobin**. *A*, deuterium exchange difference plot for PccGlobin WT in 50 mM Tris, 50 mM NaCl, pH 7.0 buffer in the physiologically relevant Fe(II)-O_2_ vs Fe(II)- unligated states. Negative differences indicate decreased exchange in the Fe(II)-O_2_ state. Standard deviations from replicate measurements are in gray. *B*, deuterium exchange differences (t = 3 min) mapped onto the homology model of PccGlobin. The heme is shown in spheres for reference but is not observed in HDX-MS spectra. Relative Deuterium uptake plot for peptides 60 to 68 (*C*) and 99 to 121 (*D*) and with (*E*) representative stacked mass spectra for peptide 99 to 121 of PccGlobin in Fe(III)-H_2_O, Fe(III)-CN, Fe(II)-O_2_, and Fe(II)-unligated states for t = 1 min and t = 3 min.

Analysis of the physiologically relevant “off” (Fe(II)-unligated) and “on” (Fe(II)-O_2_) states of *Pcc*GCS (Fig. 5, *A* and *B*) highlighted small but widespread changes in flexibility throughout the globin domain. The binding of O_2_ decreased the flexibility of distal pocket residues 52 to 56, 60 to 68 and 71 to 86 (0.89 Da at *t* = 3 min; Table S6), which is the region where the distal pocket hydrogen bond donors Y57 and S82 fall (although Y57 is not included in the covered peptides). The heme edge residue R78, which was previously shown by rR in *Pcc*GCS and X-ray crystallography in *Bpe*Globin (K64) to interact with a heme propionate (19, 25), also had decreased exchange, supporting previous studies that postulated that changes in ligation state can be propagated through the propionate (19). In addition, the proximal helix F (residues 99–121, 0.75 Da at *t* = 1 min; Fig. 5, *D* and *E*) exhibited decreased deuterium exchange differences, as compared to Fe(II)-unligated, suggesting that O_2_ binding to the heme iron decreases flexibility of the heme proximal pocket.

**Figure 6:**
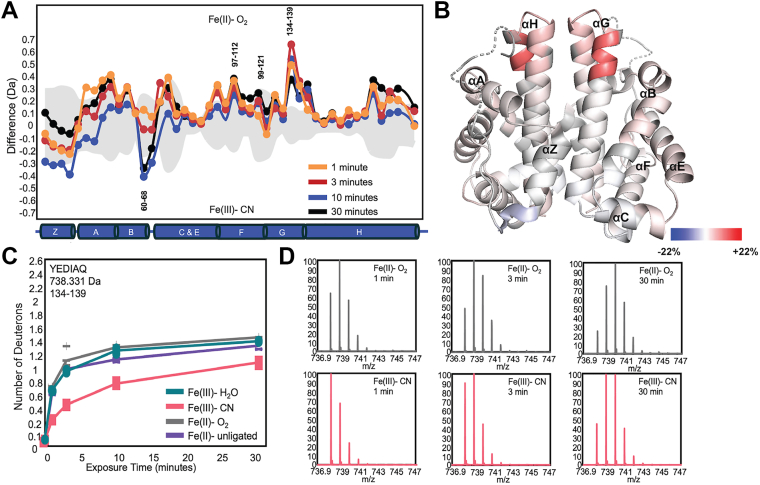
**HDX-MS Comparison of Fe(II)-O_2_ vs. Fe(III)-CN *Pcc*Globin**. *A*, deuterium exchange difference plot for *Pcc*Globin WT in 50 mM Tris, 50 mM NaCl, pH 7.0 buffer in the high activity Fe(II)-O_2_ vs. Fe(III)-CN states. Negative differences indicate decreased exchange in the Fe(II)-O_2_ state. Standard deviations from replicate measurements are in gray. *B*, deuterium exchange differences (t = 3 min) mapped onto the homology model of *Pcc*Globin. The heme is shown in spheres for reference but is not observed in HDX-MS spectra. *C*, relative deuterium uptake plot for peptides 134 to 139 (G-Helix) and (*D*) stacked mass spectra of *Pcc*Globin in the Fe(II)-O_2_ and Fe(III)-CN states at t = 1 min, 3 min, and 30 min.

Deuterium uptake differences in helices G and H, which form the globin dimer interface, were identified in residues 134 to 139 of helix G. The helix G residues at the dimer interface are ∼ 30 Å from the heme in *Bpe*Globin structures, suggesting that this region is allosterically modulated by ligand binding and may be involved in allosteric communication between the monomers. The differences in deuterium uptake in the putative allosteric site can be observed when comparing the Fe(II) vs. Fe(II)-O_2_ states, with the Fe(II)-O_2_ state exhibiting increased flexibility (0.12 Da at *t* = 10 min). These changes are more pronounced when comparing the Fe(III)-CN and Fe(II)-O_2_ states (Fig. 6, *A* and *B*; 0.54 Da at *t* = 10 min), both of which display cooperative O_2_ binding in the wild-type protein. The Fe(II)-O_2_ complex is more flexible in the allosteric region than the CN^-^ complex, with the largest difference in deuterium uptake occurring for residues 134 to 139, indicating that the CN^-^ ligand binding in the heme pocket causes the protein to adopt a more rigid conformation in helix G (Fig. 6).

## Discussion

While there are examples of cooperativity in bacterial hemoglobins, the mechanism of allostery and conservation of ligand binding cooperativity in other bacterial globins is poorly understood. The oligomeric state of bacterial sensor globins and globin coupled sensor proteins has been shown to influence O_2_ dissociation rates (21), supporting the hypothesis that oligomerization allows for communication between hemes in *Pcc*GCS. Crystal structures of the isolated globin domains from the GCS proteins *Ec*DosC (22, 43), HemAT-*Bs*, and *Bpe*GReg found that the globin domains dimerize to form a 4-helical bundle at the dimer interface (22, 23, 25). Disruption of the globin dimer interface has been shown to eliminate internal signal transduction in *Af*GcHK (36), while weakening of the dimer interface of *Pcc*Globin altered the biphasic O_2_ dissociation rates (26), suggesting that heme ligand binding in one protomer could be transduced across the 4-helix bundle to influence ligand binding in the second protomer. Previous work found that both the full-length *Pcc*GCS and *Pcc*Globin display biphasic O_2_ dissociation kinetics (21) and two midpoint potentials (39), and that oligomeric state affected O_2_ dissociation (21, 44). Biphasic equilibrium O_2_ binding has also previously been observed for human hemoglobin (30) and a bacterial GCS protein, HemAT-*Bs* (12), and was attributed to multiple heme pocket conformations of the protein existing in solution. However, unlike hemoglobin, HemAT-*Bs* did not exhibit positively cooperative O_2_ binding and cooperativity in other sensor globins has not been reported to date.

*Pcc*GCS was used as a model to probe ligand binding cooperativity, and equilibrium binding measurements showed positively cooperative homotropic ligand (O_2_/CN^-^) binding in WT *Pcc*GCS and the isolated globin domain, *Pcc*Globin (Table 1). These results highlight that globin dimerization is sufficient for cooperative ligand binding and that, while the middle and cyclase domain contacts are important to DGC activity (45), they are not required to transduce ligand binding cooperativity between the globin domains. However, similar to what has been seen in hemoglobin (38), conformational changes linked to cooperative O_2_/CN^-^ binding by *Pcc*GCS do not appear to directly correlate to shifts in the redox potential (Table S2). However, the distal tyrosine residue (Y57) is required to observe two midpoints and therefore has a role in modulating heme midpoint potential(s) without being involved in redox cooperativity. Perturbations to the distal heme pocket of GCS proteins have previously been shown to modulate ligand binding stability (26). *Ec*DosC, the globin-coupled DGC from *E*. *coli*, naturally lacks the second distal pocket hydrogen bond donor (43) and has a single, high O_2_ dissociation constant (20, 43), supporting the role of hydrogen bond donors in modulating O_2_ binding. Unlike *Pcc*Globin WT, the *Pcc*Globin S82A variant also only exhibits a single O_2_ dissociation rate (26) and does not cooperatively bind O_2_ (Table 1), suggesting that two hydrogen bond donors are required in the heme distal pocket to allow for cooperative O_2_ binding. Given the loss of O_2_ binding cooperativity for the S82A variant, the retention of CN^-^ binding cooperativity (Table 2) suggests that Fe(II)-O_2_ vs. Fe(III)-CN ligation states have slight differences in contacts within the heme pocket and that hydrogen bonding is strongly tied to O_2_ binding cooperativity.

As observed by rR, spectra of Fe(III)-CN *Pcc*Globin WT, S82A and Y57F exhibit quite similar rR spectral patterns, with the heme skeletal modes and out-of-plane distortions remaining unchanged (Fig. 2, *B*, *D*, and *F*). These findings indicate that mutating these key residues does not significantly induce conformational changes in the heme of the Fe(III)-CN complex. This consistency in rR stretching modes also demonstrates that interactions with the heme and bound CN^-^ ligand are maintained in the absence of distal pocket hydrogen bonding side chains. Conservation of the H-bonding network near the CN^-^ ligand in the WT and variant proteins is consistent with the retention of CN^-^ cooperativity in the *Pcc*GCS S82A mutant (46) (58). The similarities in the Fe(III)-CN structures of *Bpe*Globin WT vs. S68A and the rR spectra of Fe(III)-CN *Pcc*Globin WT vs. S82A suggest that the bound CN^-^ can induce changes in protein/heme conformation even in the absence of the distal hydrogen bonding network.

The differences in heme pocket interactions for Fe(II)-O_2_ ligation states of *Pcc*GCS residue variants, as compared with the minimal changes in the Fe(III)/Fe(III)-CN states, suggests that heme deformation, heme propionate interactions, and hydrogen bonding to the bound O_2_ are required for cooperative O_2_ binding (Fig. 3). Previous work has suggested that heme flattening is important for signal transduction in both the H-NOX family (46, 47) and GCS signal transduction (19). As the S82A variant exhibits flattening of the heme macrocycle in the Fe(II)-O_2_ ligation state, as well as increased affinity for O_2_ and loss of O_2_ binding cooperativity (Table 1, S5), the rR data suggest that heme flattening results in significant conformational changes in the *Pcc*Globin heme pocket. In addition, alterations to the geometry of heme peripheral groups are observed in S82A mutant, with the mutation causing an activation of the bending modes of propionate groups (368 and 375 cm^−1^), indicating structural reorientation and perturbed hydrogen-bonded propionate groups (Fig. 3*B*).

The shift in the ν(Fe-O) mode for the S82A variant compared to the WT is likely associated with a non-hydrogen bonded O_2_ form (29), indicating that the S82A mutation induced a conformational change which interrupted H-bonding with heme-bound O_2_, supporting the hypothesis that the H-bonding network is required for cooperative O_2_ binding. A related globin coupled sensor, HemAT-*Bs*, exhibits three different conformers in the wild-type O_2_-bound forms, with rR spectra showing oxygen isotope-sensitive bands at 554, 566, and 572 cm^-1^, designated as the closed form, open α form, and open β form, respectively (29). These varied frequencies were interpreted to represent different hydrogen bonding interactions in the distal pocket of HemAT-*Bs*. The low-frequency ν(Fe-O) at 554 cm^-1^ is linked to a strongly hydrogen-bonded Fe-O-O fragment, mediated by a water molecule between O_2_ and T95, the middle frequency ν(Fe-O) at 566 cm^-1^ indicates a moderately strong hydrogen bond between the Fe-O-O fragment and a nearby amino acid residue, and the highest frequency ν(Fe-O) at 572 cm^-1^ corresponds to a conformer with no hydrogen bond interaction with O_2_. Mutation of T95 A in HemAT-*Bs* eliminates the closed and open α forms, indicating that T95 residue is involved in the hydrogen bonding interaction with O_2_ and thus required for O_2_ sensing in signal transduction. Considering that the S82 residue in *Pcc*GCS is located at the same position as T95 residue in HemAT-*Bs* (23), our rR data provide definitive evidence that this distal residue acts as a key hydrogen bond donor. The replacement of S82 with alanine effectively disrupted the H-bonding network in the heme distal site, resulting in a non-hydrogen bonded Fe-O-O fragment. This disruption in hydrogen bonding impacts the O_2_ dissociation rate, but doesn’t affect the extremely slow autoxidation rate (21, 26), and is involved in loss of allosteric communication within the globin domain for the *Pcc*Globin S82A variant.

Hydrogen bonding of heme propionates with heme-edge residues, such as R78, was previously shown to be linked to heme ligation/oxidation state in *Bpe*Globin and is required for O_2_-dependent activation of the cyclase domain, likely due to the induction of significant conformational changes in the heme active site (19). Furthermore, the differences in heme deformation underscore the role of subtle changes in heme pocket interactions in ligand-binding cooperativity (Table S5). Together, these data suggest that small changes in the hydrogen bonding network around the heme upon ligand binding are propagated through the entire protein through subtle rearrangements in the helical positions, allowing for communication between heme domains.

To elucidate the mechanism of ligand-binding cooperativity, *in crystallo* and solution state experiments were employed to understand the conformational dynamics of the ligand binding pocket and identify potential allosteric sites. Crystallization of the *Pcc*Globin homolog, *Bpe*Globin, in the Fe(III)-CN state showed minimal structural differences (Fig. 4*D*), as compared to the Fe(II)-O_2_ state previously crystalized, supporting the hypothesis that ligand specific interactions are propagated through subtle changes in electrostatic contacts, rather than through large structural rearrangements (25). Furthermore, the *Bpe*Globin WT and heme pocket serine variant Fe(III)-CN crystal structures also showed strong structural similarity (Fig. 4, *B* and *C*), supporting the hypothesis that allostery communication occurs through dynamic interactions, rather than through significant structural changes. While comparison of structures in various ligation states and variants did not provide new insights into signaling, it highlighted the need to probe the conformational changes of *Pcc*Globin in different ligation and oxidation states using HDX-MS.

Previous studies on *Pcc*GCS have found that removing the linkage between the proximal histidine and proximal helix (through mutation of His- > Gly and addition of imidazole to serve as the fifth ligand to iron) decreases allosteric communication from the globin to diguanylate cyclase domain, supporting a role for proximal helix flexibility in signaling (18). Overall, the observation that *Pcc*GCS Fe(II)-unligated exhibits the greatest deuterium uptake in the proximal pocket (helix F, residues 99–121) suggests that O_2_ binding results in the formation of hydrogen bonding networks that stabilize the heme pocket, allowing for allosteric communication (Fig. 5, *A* and *B*). Decreased dynamics and/or conformational changes of the proximal helix upon ligand binding have been observed for other heme proteins due to the interaction between the bound ligand and both heme iron and distal pocket residues provides a direct linkage between the proximal and distal halves of the protein (24).

**Figure 7:**
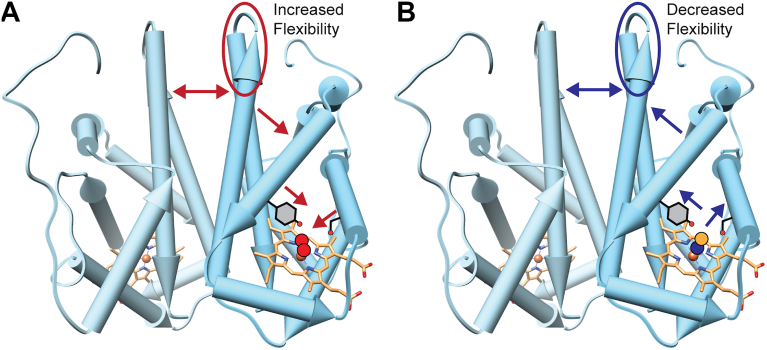
**Model of allosteric signaling**. *A*, O_2_ binding results in hydrogen bonding interactions that decrease heme pocket and B helix flexibility, resulting in additional flexibility of helices G and H, which is transmitted across the dimer interface. In contrast, binding of CN^-^ (*B*) results in steric interactions that are propagated to decrease G/H helix flexibility, which also can be transmitted across the dimer interface. The changes in G/H helix flexibility are proposed to be sufficient to propagate allosteric communication and allow for cooperative ligand binding.

Identification of changes in flexibility in helix G residues at the dimer interface ∼ 30 Å from the heme in *Bpe*Globin structures suggests that this region is allosterically modulated by ligand binding and may be involved in allosteric communication between the monomers (Fig. 6*B*). While *Pcc*Globin WT exhibits cooperative binding for both O_2_ and CN^-^, the Fe(II)-O_2_ complex is more flexible in the allosteric region than the Fe(III)-CN complex. In contrast, residues 60 to 68, which span helix B and C within the distal heme pocket, show decreased flexibility in the Fe(II)-O_2_ state, as compared to the Fe(III)-CN state, further supporting the importance of specific hydrogen bonding contacts in the distal heme pocket in ligand discrimination. Similar heme oxidation state-specific responses in flexibility were observed for *Af*GcHK in the distal pocket region (21), supporting the results observed for *Pcc*Globin. Taken together, these data suggest a model of signal transduction in which ligand binding to the heme pocket causes changes that are dependent on ligand identity, altering dynamics of inter-helix contacts, and finally changing the flexibility of helices G and H, which is propagated across the dimer interface (Fig. 7).

Overall, this work suggests a mechanism of signal transduction wherein ligand binding alters heme pocket interactions and heme conformation, which results in broad changes in dynamics throughout the protein, including changes in conformation of the distal heme pocket and the proximal helix. The crystal structures of *Bpe*Globin WT and S82A, rR spectra of *Pcc*GCS variants, and HDX-MS of *Pcc*Globin indicate that the heme conformation and distal hydrogen bonding network are required for O_2_ binding to form key hydrogen bonding interactions that are propagated throughout the protein, changing helix G flexibility and allowing for cooperative O_2_ binding. In contrast, cooperative CN^-^ binding does not require distal pocket hydrogen bonding interactions, as evidenced by the lack of differences in the X-ray structures and rR spectra of distal pocket variants, suggesting that the changes in distance between the heme and distal pocket are sufficient to elicit the conformational changes required to transduce ligand binding signal across the dimer interface.

## Conclusion

Results of these studies demonstrate that bacterial GCS proteins and sensor globins can exhibit cooperative ligand binding, but O_2_-dependent allosteric communication between hemes requires specific heme pocket interactions. Distal pocket hydrogen bonding residues that interact with bound ligands play important roles in recognizing the ligand identity and transmitting the ligand binding event between heme domains. The relative polarity of the *Pcc*GCS heme pocket, as compared to HemAT-*Bs* (48), may contribute to the positive ligand binding cooperativity. Additionally, while CN^-^ is often used as an O_2_ mimic to preclude heme autooxidation that is often observed for Fe(II)-O_2_ complexes, this work has shown that heme pocket mutations have different effects on O_2_ vs. CN^-^ binding cooperativity and heme conformation, suggesting potential challenges with using the Fe(III)-CN state as a mimic in heme proteins.

Taken together, this work provides new insights into the mechanism of ligand binding cooperativity and allosteric signaling mechanisms in multi-domain proteins. As GCS proteins are involved in regulating responses to environmental signals and are important for behaviors such as biofilm formation and virulence, cooperative ligand binding may allow the bacteria to respond robustly to small changes in O_2_ tension and rapidly alter their physiology. Given the importance of allostery in modulating both ligand affinity and signaling, the pathways identified in these studies provide an opportunity to expand our understanding of ligand binding to heme proteins and signal transduction in bacterial sensing proteins.

## Experimental procedures

### Protein expression and purification

*Pcc*GCS/*Bpe*GReg (21) and *Pcc*Globin/*Bpe*Globin (26) WT and globin domain serine and tyrosine variants were isolated and purified as previously described. All proteins were heterologously expressed in *E*. *coli* DE3 Tuner *plysS* (Novagen) competent cells in expression media containing 45 g/L yeast extract, 1% v/v glycerol and 100 mM potassium phosphate buffer, pH 7.95 with 100 mg mL^-1^ ampicillin and 30 μg mL^-1^ chloramphenicol. Cultures were grown at 37 °C until the OD_600_ reached 0.7, at which time protein expression was induced with 100 μM IPTG and 500 μM aminolaevulinic acid. The temperature was lowered to 20 °C at induction and expression of full-length *Pcc*GCS and *Bpe*GReg continued for 6 h or 20 h for the truncated globin domains. The cultures were harvested by centrifugation at 4000 x g at 4 °C for 20 min. The resulting pellet was stored at −80 °C until use.

Protein purification was done at 4 °C. Cell pellets were resuspended in 50 mM Tris-HCl, 300 mM NaCl, 20 mM imidazole, 5% v/v glycerol pH 7.4 with benzamidine and PefaBloc SC to inhibit protease cleavage. The cells were lysed using sonication (30 s on/30 s off, 6 min, 60% amplitude) and centrifuged at 130,000 x g in a Beckman Optima L-90X ultracentrifuge at 4 °C for 1 h. The supernatant was loaded on a HisPur Ni-column (Fisher Scientific) and eluted with 50 mM Tris, 300 mM NaCl, 250 mM imidazole, 5% v/v glycerol pH 7.4. Proteins were further purified with a S200 gel filtration column (GE Healthcare) equilibrated with 50 mM Tris, 50 mM NaCl, 1 mM Dithiothreitol (DTT) (Research Product Int.), 5% glycerol v/v, pH 7.0. Fractions were concentrated through ultrafiltration (10 kDa MWCO filter, Millipore), flash frozen, and stored at −80 °C until use.

### Equilibrium ligand measurements

Equilibrium O_2_ binding was studied through direct titration of oxygenated buffer (50 mM Tris, 50 mM NaCl, pH 7.0) solutions in airtight cuvettes (Starna Inc) with minimal headspace to reduce pressure effects on O_2_ solubility. The protein of interest was reduced in an anaerobic chamber (Coy Laboratories) using sodium hydrosulfite, desalted on a PD-10 column with anaerobic buffer (50 mM Tris-HCl, 50 mM NaCl, pH 7.0), then diluted to a final concentration of 500 nM. Buffer oxygen concentrations were measured with a fiberoptic oxygen mini-sensor (Pyroscience, OXR50). Buffer was delivered to the sealed cuvette using a gas-tight syringe (Hamilton) at room temperature. Changes in the absorbance were measured using an Ocean HDX miniature spectrometer (Ocean Optics) in the anaerobic chamber. Data was fit to the Hill equation using Igor Pro (Wavemetrics), where *θ* represents to the fraction of the population bound to O_2_ measured by the ratio of the Soret band for the heme Fe(II)-O_2_ species at 415 nm to the Fe(II)-unligated species at 434 nm (1), L represents the concentration of oxygen in the sample, K_d_ represents the dissociation constant, and n represents the Hill constant. Error measurements were calculated by propagating the error in the triplicate titrations performed for each protein variant. Representative spectra are shown in Figure S1, with the fitting for the Hill equation and residuals.

(1) $$\theta ={\left[L\right]}^{n}/\left(K_{d}+{\left[L\right]}^{n}\right)$$

Equilibrium CN- binding was measured using direct titrations of potassium cyanide solution to airtight cuvettes in the same manner as the O_2_ titrations. Purified protein was chemically oxidized with potassium ferrocyanide, desalted on a PD-10 column with aerobic buffer (50 mM Tris-HCl, 50 mM NaCl, pH 7.0). Potassium cyanide was dissolved in aerobic buffer and delivered to the sealed cuvette with a gas-tight Hamilton syringe 500 nM protein in aerobic buffer. Absorbance was measured using a Cary100 UV-vis spectrometer (Agilent) at the Soret band for the heme Fe(III)-CN species at 420 nm and the Fe(III)-unligated species at 400 nm (1), to determine the fraction of the sample bound to CN^-^, and fit with Igor Pro (Wavemetrics) to Equation 1, with L as the concentration of CN^-^ in the final cuvette.

### Redox properties of *Pcc*GCS

Midpoint potentials of *Pcc*GCS were measured as previously described (39). Electrochemical titrations were performed in an optically transparent thin-layer cell (OTTLE) purged with argon to create an anaerobic environment (49). Proteins were either chemically oxidized with potassium ferrocyanide or reduced with sodium hydrosulfite, desalted on a PD-10 column with 50 mM HEPES, (Research Products Int.) 50 mM KCl (Research Products Int.), pH 7.0 buffer, then mixed with 1X of 1000X mediator stock solutions. Mediators (all purchased from Sigma-Aldrich) in the stock solution were 20 mM duroquinone, 10 mM pyocyanin, 10 mM 2-hydroxy 1,4- naphthoquinone, 10 mM anthraquinone-2-sulfonate, 2 mM benzyl viologen, 1 mM phenosafranin, and 1 mM indigo carmine. All mediators were dissolved in degassed DMSO (Sigma-Aldrich) except for benzyl viologen, which was dissolved in anaerobic water.

A solution of ∼400 μM *Pcc*GCS and 1X mediator mix (in 50 mM HEPES, 50 mM KCl, pH 7.0) was introduced to the purged OTTLE cell with a Hamilton syringe. Controlled-potential coulometry of the OTTLE cell was achieved using a three-electrode potentiostat (Chi-CH Instrument Electrochemical Analyzer). The electronic potentials of the sample were maintained by coulometric generation of mediator-titrant, and the temperature of the sample was maintained at 25 °C using an Agilent Technologies Cary Temperature Controller. Midpoint potentials are reported relative to the standard hydrogen electrode (SHE). Fitting analysis was performed using Igor Pro (Wavemetrics) with one or two midpoint potentials as previously described (39).

Analysis of the *Pcc*GCS Soret bands under varying potential was conducted. The log of fraction of the population [oxidized]/[reduced] using the 434 nm Soret band was plotted as a function of the potential and fit to the Nernst equation (Equation 2) as previously described (50). Cooperativity was determined from the first derivative of the Nernst plot, where E is the potential of the working electrode, E^o^ is the reduction potential when log[ox]/[red] = 0, n is the slope, and 59.2 is the value of RT/F at 25 ^°^C.

(2) $$\log\left[\text{ox}\right]/\left[\text{red}\right]=\left(n/59.2\right)E-\left(n/59.2\right)E^{o}$$

### HDX-MS

HDX-MS experiments probed the solution state dynamics of different ligation and redox states. *Pcc*Globin samples were prepared in the ferric cyano, ferric aqua, ferrous oxy and ferrous unligated states in 50 mM Tris-HCl, 50 mM NaCl, pH 7.0 as previously described. Proteins were diluted 20-fold in a deuterium labeling buffer (95% D_2_O, 50 mM Tris-HCl 50 mM NaCl, pH 7.0) to a final concentration of 0.5 μM protein in 90% D_2_O and a total reaction volume of 60 μl. The deuterium exchange reaction was carried out in triplicate at 20 °C for 1-, 3-, 10-, and 30-min labeling times using a PAL-CTC autosampler (Leap). Two biological replicates of each ligation state, with three technical replicates each, were used to validate the results. The reaction was quenched by dropping the pH to 2.5 with the addition of cold 60 μl 1.5 M guanidinium hydrochloride, 0.25 M Tris(2-carboxyethyl)phosphine hydrochloride (TCEP).

Quenched samples were then injected into an ACQUITY nano-UPLC HDX sample manager (Waters Inc) and digested in an immobilized BEH pepsin column (2.1 x 30 mm) (Waters, USA) with a continuous flow rate of 75 μl/min in 0.1% formic Acid. The resulting peptides were trapped in a trap column (Acuity BEH C18 VanGuard pre-column) (Waters Inc), loaded onto an ACQUITY BEH C-18 reverse-phase chromatography column (Waters Inc) and eluted under an 8%–40% gradient of acetonitrile in 0.1% formic acid at 40 μl/min pumped from an ACQUITY Binary Solvent Manager (Waters Inc). The peptides were ionized by electrospray ionization and analyzed in a Synapt XS mass spectrometer (Waters Inc) in the HD-MS^E^ mode. The mass spectrometer was calibrated continuously with 100 fmol/μl Glu-Fibrinopeptide B at a flow rate of 5 μl/min. The total run time of LC-MS was 15 min, consisting of a 3-min pepsin digestion step and 12 min of separation and acquisition.

Deuterium exchange was analyzed using DynamX v3.0 (Waters Inc). The peptides were filtered with cutoffs listed: minimum intensity: 5000, minimum peptide length: 5, maximum peptide length: 25, mass tolerance: 10 ppm, minimum products:1, and file threshold: 2. Deuterium exchange values (Da) were determined from the difference of the centroid of the deuterated mass envelopes compared to the centroids of corresponding undeuterated mass envelopes. Deuterium exchange profiles comparing two different states were plotted in difference plots and mapped onto the *Pcc*Globin homology model in PyMOL.

### Crystallography

*Bpe*Globin was overexpressed and purified as described above. Purified *Bpe*Globin was oxidized in the presence of 1 mM potassium cyanide. The Fe(III)-CN protein complex was isolated using a PD-10 column, and the ligation state was confirmed using UV-vis spectroscopy (spectra covered 250–700 nm, encompassing the Soret and Q-bands, which are indicative of ligation/oxidation state). The protein was then concentrated to 5 mg/ml using 10 kDa MWCO spin concentrators (Millipore-Sigma) in 50 mM Tris (pH 7.0), 50 mM NaCl and UV-vis spectroscopy was used to verify that the CN^-^ ligand remained bound. Protein samples were crystallized by using the sitting drop vapor diffusion technique at room temperature. Crystals were obtained after 2 days by mixing a 1 μl drop of protein with 1 μl of precipitant solution containing 10 mM Tris (pH 8.0), 0.2 M calcium acetate, and 10% w/v PEG 3350. Crystals were harvested in rayon loops and cryoprotected in the well solution supplemented with 20% glycerol prior to flash freezing in liquid nitrogen. Both WT *Bpe*Globin and S68 A *Bpe*Globin were analyzed under the same crystallization conditions and cryoprotection strategies.

Diffraction data for wild-type *Bpe*Globin Fe(III)-CN were collected at LS-CAT sector 21 at the Advanced Photon Source at Argonne National Laboratory. The S68A *Bpe*Globin Fe(III)-CN diffraction data were collected at beamline 17 of the National Synchrotron Light Source II at Brookhaven National Laboratory. The HKL2000 software package was used to index, integrate, and scale all datasets (51). The structures were solved using molecular replacement (MR), performed with Phaser (52) implemented in the CCP4 package (53), with the structure of oxy-ligated WT *Bpe*Globin as the search model (PDB accession code 6m9a). Model building was performed in Coot (54), and refinement was carried out using Refmac5((55)) at the initial stages and Phenix (56) for final refinements. Figures were created using Chimera (57).

Wild-type *Bpe*Globin Fe(III)-CN and S68A *Bpe*Globin Fe(III)-CN crystallize in the C_1_2_1_ space group with three monomers in the asymmetric unit. The final model of wild-type *Bpe*Globin Fe(III)-CN is comprised of residues 14 to 168 in chain A, residues 14 to 168 in chain B, residues 14 to 167 in chain C, a heme and bound cyanide ligand, and 112 water molecules. The final model of S68 A *Bpe*Globin Fe(III)-CN S68A consists of residues 13 to 168 in chain A, residues 13 to 168 in chain B, residues 14 to 168 in chain C, a heme and bound cyanide ligand per monomer, and 110 water molecules.

Evidence of photoreduction was observed in the structures. Photoreduction of ferric heme complexes in X-ray crystallography has been observed in many well-characterized ferric heme complexes, including myoglobin, hemoglobin, and cytochrome450cam (58, 59, 60). CN^-^ binds to the iron in linear geometry with an Fe-C-N angle between 160 to 180 degrees. In myoglobin, the elongation of the Fe-CN bond and the angle of the CN^-^ ligand have been used to demonstrate photoreduction of histidyl ligated ferric heme complexes. Reduction of the iron from Fe(III) to Fe(II) causes the CN^-^ ligand affinity to decrease and the bond length to increase (61). However, recent work on the peroxidase from *Klebsiella pneumoniae*, *Kp*DyP, has shown that analysis of the electron density is an important component of assigning photoreduction (62), given that the CN^-^ ligand is manually restrained during refinement, so the bond length is subject to experimental bias.

While the Fe-CN bond lengths of all three subunits of *Bpe*Globin WT and S68A fall within the 1.49 to 2.31 Å range of Fe-CN bond lengths in reported GCS proteins, HemAT*bs* (23) and *Af*GcHK(26), and sperm whale myoglobin (59) (Table S2), the electron densities show deviation from anticipated geometry of the complex. In WT *Bpe*Globin Fe(III)- CN, the bond lengths of all three chains for both the proximal histidine and CN^-^ ligand are similar, but the angle of the CN^-^ ligand (160.5 degrees) is closest to the reported values for HemAT*bs* (1OR4) and sperm whale myoglobin (1EBC) in chain A (164–173 degrees). In the S68A variant, the bond lengths are within the range of other histidyl-ligated ferric cyano heme complexes, but additional density around the CN^-^ ligand suggests photoreduction occurred in all three chains, with chain B showing the least evidence of photoreduction (Figs. X, SI). Subunits with the least evidence of photoreduction were chosen to analyze the conformational change in both WT *Bpe*Globin Fe(III)- CN and S68 A *Bpe*Globin Fe(III)- CN. Therefore, Chains A in WT *Bpe*Globin and chain B in the S68A variant were used for the subsequent structural comparisons.

The X-ray crystallography data and structures were deposited in the Protein Data Bank (www.rcsb.org; WT, PDB: 9DSE; S68A, PDB: 9DSF).

### Resonance raman

#### Sample preparation

Resonance Raman (rR) spectroscopy was employed to analyze alterations in heme conformation and properties in *Pcc*GCS WT full length, *Pcc*Globin, and *Pcc*Globin S82A variants. To prepare the ferrous unliganded states (wild type and S82 A mutant), each sample containing 100 μl of 100 μM protein in 50 mM Tris, 50 mM NaCl, 1 mM DTT, pH 7.0 and 5% glycerol v/v was transferred to NMR tubes (WG-5M-ECONOMY-7, Wilmad Glass Co., Beuna, NJ). The deoxy samples were generated by evacuation of the samples on a vacuum line and then refilling with argon gas three times. Sufficient dithionite solution was added to fully reduce the samples, as confirmed by monitoring the Q region of electronic absorption spectrum, using a device (ISS-2 integrated sampling system, Ocean Optics Inc) that can measure spectra for the NMR tubes connected to vacuum line. To prepare the oxygenated samples, approximately 10 ml of oxygen gas (^16^O_2_ or ^18^O_2_) was added with a gas tight syringe to the ferrous *Pcc*GCS solutions to form the oxy complexes. The samples were gently shaken for 10 s followed by rapid freezing in a liquid nitrogen bath. To prepare the *Pcc*GCS ferric CN complexes, each sample was oxidized by adding potassium ferrocyanide solution and the excess KFe(CN)_6_ was removed from *Pcc*GCS samples by passing through a PD10 desalting column (GE Healthcare) with aerobic buffer (50 mM Tris-HCl, 50 mM NaCl, pH 7.0). Oxidized protein was incubated with excess KCN for 30 min and desalted again through a PD10 column with aerobic buffer, as described above. The ligation state was confirmed using UV-Vis spectroscopy. The protein was finally concentrated to 100 μM for rR measurements.

#### rR measurement

The rR spectra of ferrous forms were acquired with the 441.6 nm line from a He-Cd laser (1K Series He-Cd laser, Kimmon Koha., Ltd), while the ferric aqua, ferric cyano complexes and oxygenated samples were measured using the 413.1 nm excitation line, which is from a Kr + laser (Coherent Innova Sabre Ion Laser). All spectra were measured using a Spex 1269 spectrometer equipped with a Spec-10 LN liquid nitrogen-cooled detector (Princeton Instruments, NJ). The rR spectra were collected using back scattering (180°) geometry with the laser beam being focused by a cylindrical lens to form a line image on the sample to help prevent heating and minimize photodissociation. The laser power incident on the ferrous, ferric and ferric-CN samples was maintained at ∼10 mW, while being adjusted to 1 mW or less on the oxygenated samples. The slit width was 150 μm, and the 1200 g/mm grating was used for measurements. Spectra were calibrated with data acquired for fenchone and processed with Grams/32 AI software (Galactic Industries, Salem, NH).

## Data availability

X-ray structures have been uploaded to the Protein Data Bank as 9DSE and 9DSF.

## Supporting information

This article contains supporting information (6,22, 23, 24, 25,36,37,64).

## Conflict of interest

The authors declare that they have no conflicts of interest with the contents of this article.
