# Supplementary material for: Cooperative ligand binding in a bacterial heme-based oxygen sensor
Source: J Biol Chem. 2025 Dec 8;302(2):111025. doi: 10.1016/j.jbc.2025.111025 (PMC12805357; doi:10.1016/j.jbc.2025.111025)
Supplement: Supplementary Material 1 [file mmc1.pdf]

Cooperative Ligand Binding in a Bacterial Heme-Based O_2 Sensor

Nushrat J. Hoque, Sarah R. Pope, Varun Venkatakrishnan, David O. Olori, Noah A. Brady,
Dayna C. Patterson, Ganesh S. Anand, Yilin Liu, Amie K. Boal, and Emily E. Weinert

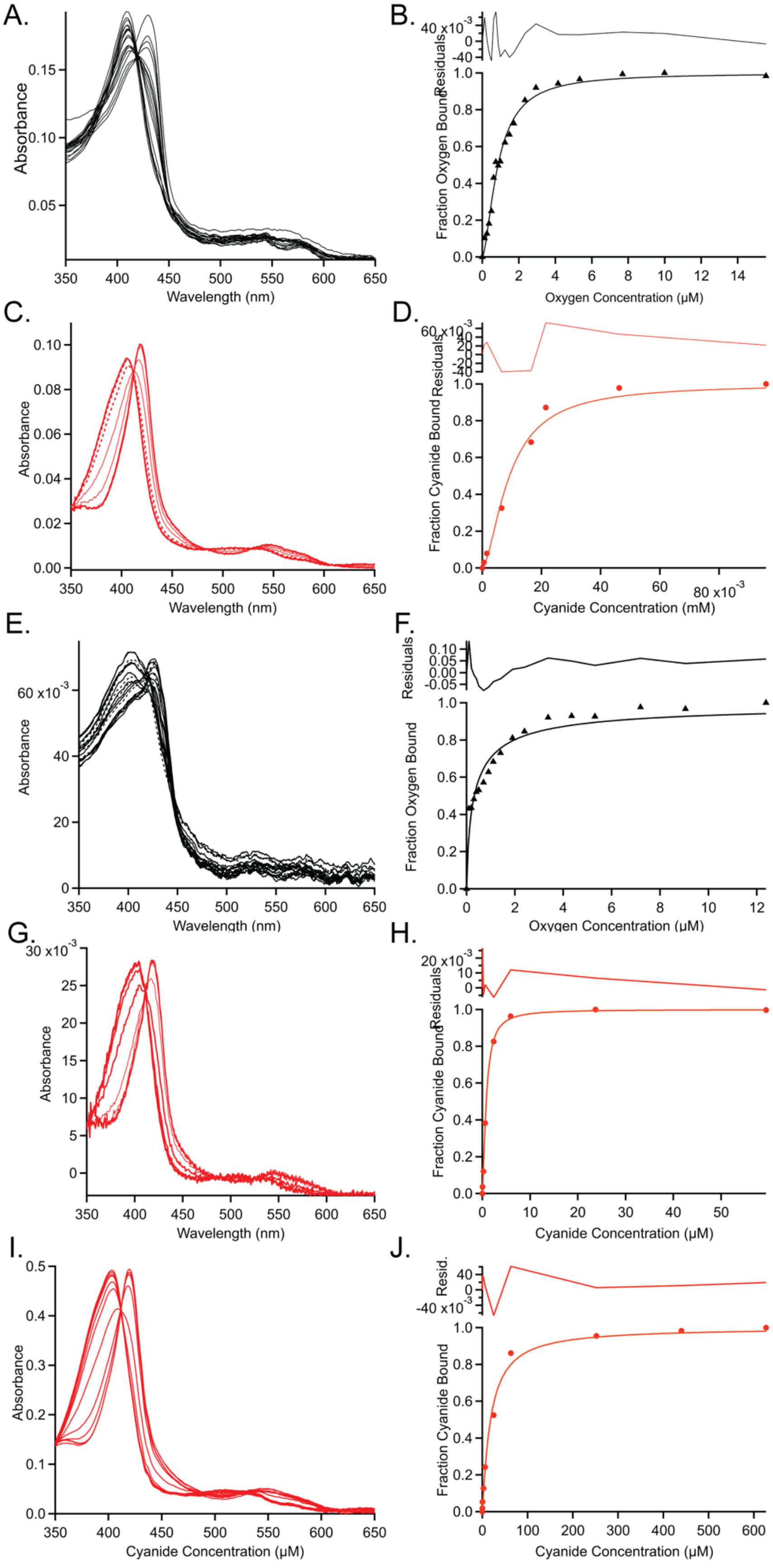
A.
Absorbance
Wavelength (nm)
B.
Residuals
Fraction Oxygen Bound
Oxygen Concentration (µM)
C.
Absorbance
Wavelength (nm)
D.
Residuals
Fraction Cyanide Bound
Cyanide Concentration (mM)
E.
Absorbance
Wavelength (nm)
F.
Residuals
Fraction Oxygen Bound
Oxygen Concentration (µM)
G.
Absorbance
Wavelength (nm)
H.
Residuals
Fraction Cyanide Bound
Cyanide Concentration (µM)
I.
Absorbance
Cyanide Concentration (µM)
J.
Resid.
Fraction Cyanide Bound
Cyanide Concentration (µM)

Figure S1: Representative Spectra of *Pcc*Globin Equilibrium O_2 and CN^- Titrations. (A) Spectra of titration of oxygenated buffer (50 mM Tris, 50 mM NaCl, pH 7.0) into *Pcc*Globin WT. (B) Data from (A) fit to the Hill equation in Igor. Residuals for fitting are shown above the Hill plot. (C) Absorbance spectra of *Pcc*Globin WT titrated with KCN. (D) Data from (B) fit to the Hill equation in Igor. Fit residuals for fitting are shown above the Hill plot. (E) Absorbance spectra of *Pcc*Globin S82A titration with oxygenated buffer. (F) Data from (E) fit to the Hill Equation in Igor. Residuals for fitting are shown above the Hill plot. (G) Absorbance spectra from KCN titration into *Pcc*Globin S82A. (H) Data from (G) fit to the Hill Equation in Igor. Residuals for fitting are shown above the Hill plot. (I) Absorbance spectra from KCN titration into *Pcc*Globin Y57F. (J) Data from (I) fit to the Hill Equation in Igor. Residuals for fitting are shown above the Hill plot.

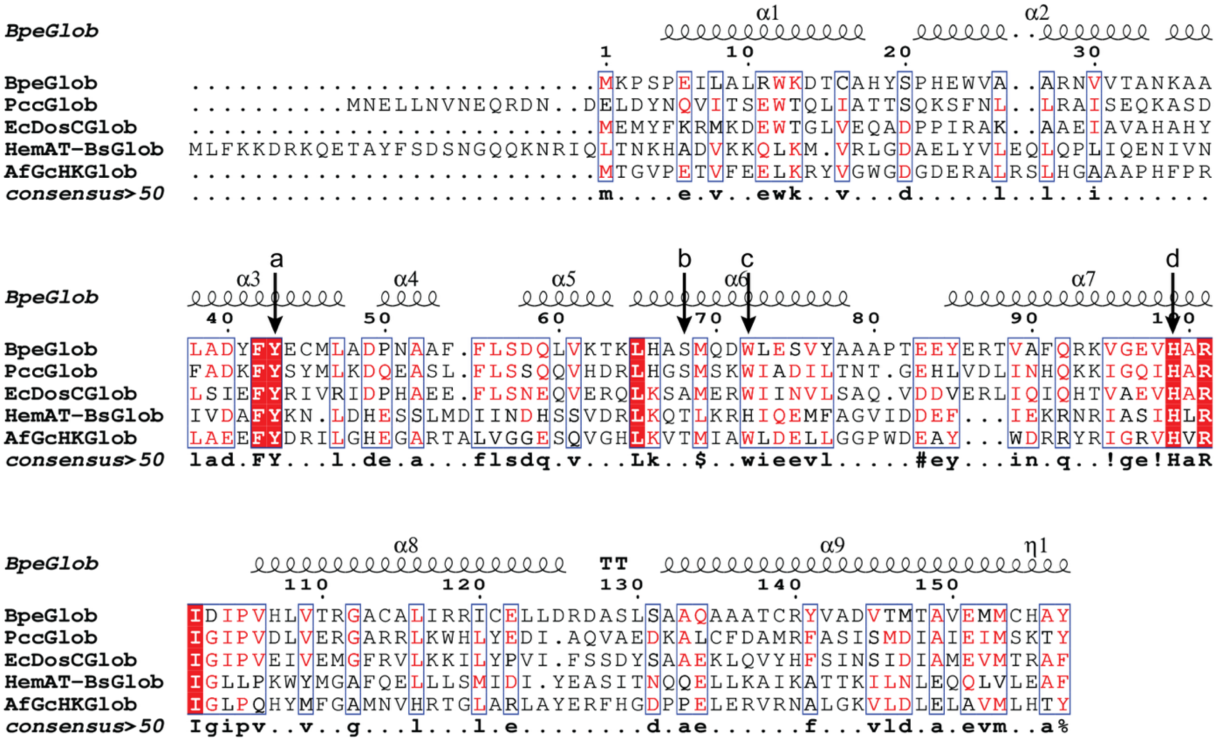

Figure S2: Globin sequence alignment of *Pcc*Globin and crystallized sensor globins. Sequence alignment of *Bpe*Globin, *Pcc*Globin, *Ec*DosC globin, HemAT-*Bs* globin, and *Af*GcHK globin with the distal tyrosine (a), distal serine (b), heme edge residue (c), and proximal histidine (d)highlighted with arrows. *Bpe*Globin has residues identical to those in *Pcc*Globin in previous identified key heme pocket positions, while the other crystallized sensor globins[1–5] have variations in the distal serine and heme edge positions.

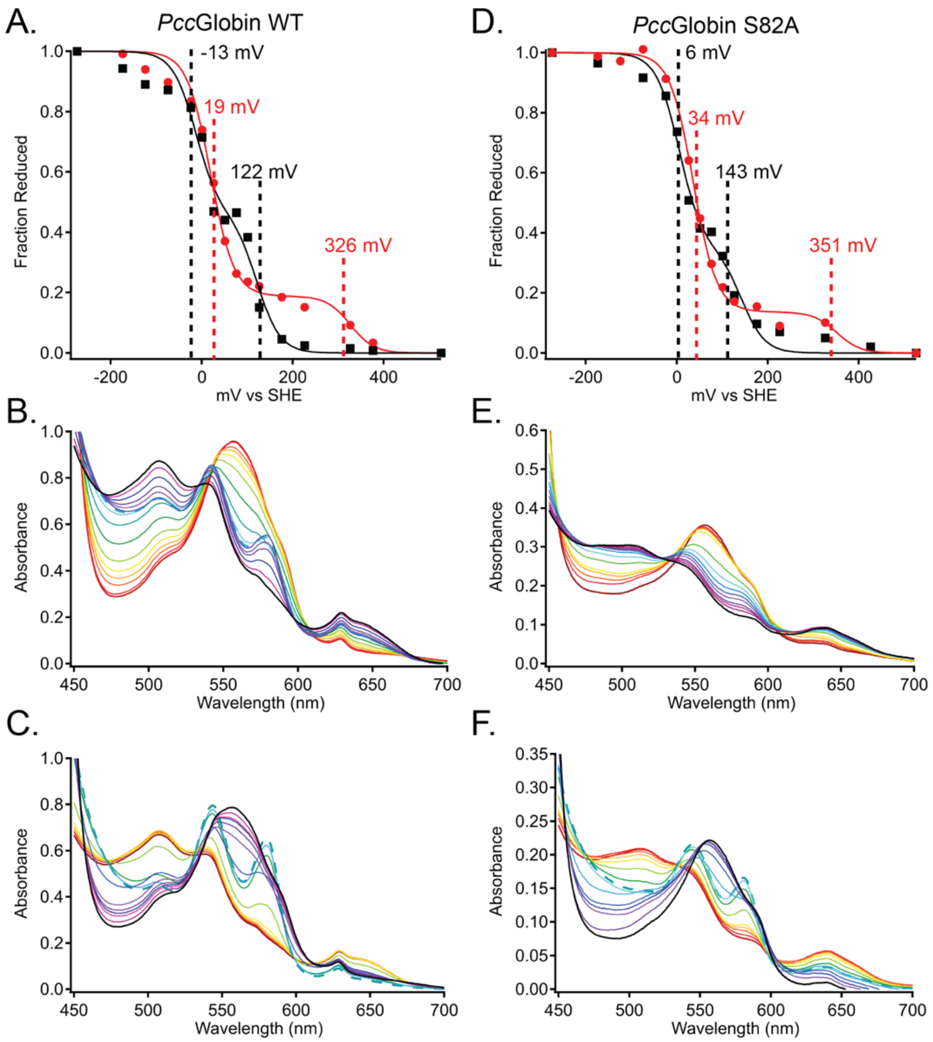

Figure S3: **Electrochemical characterization of *Pcc*Globin WT and *Pcc*Globin S82A variant.** (A) Representative titration of *Pcc*Globin WT in 50 mM HEPES, 50 mM KCl, pH 7.0. The oxidative titration (red) and reductive titration (black) midpoint potentials were determined using the spectral changes at 640 nm and plotting the fraction of reduced iron against the set potential. The data was fit as previously described to two, reversible redox active components.[6] (B) Absorption spectra of *Pcc*Globin WT from oxidative titration in an OTTLE cell. UV-visible spectra were recorded for each potential. Multiple isosbestic points indicate the presence of more than 2 species in solution. (C) Absorption spectra of *Pcc*Globin WT from reductive titration in an OTTLE cell. Splitting of the Q band (blue dashed line) indicates binding to the heme iron may be occurring. (D) Representative titration of *Pcc*Globin S82A in 50 mM HEPES, 50 mM KCl, pH 7.0. The oxidative titration (red) and reductive titration (black) midpoint potentials were determined using the spectral changes at 640 nm and plotting the fraction of reduced iron against the set potential. The data was fit to two redox active components.[6] (E) Absorption spectra of *Pcc*Globin S82 from oxidative titration in an OTTLE cell. (F) Absorption spectra of *Pcc*Globin S82A from reductive titration in an OTTLE cell. Splitting of the Q-band (blue dashed line) is indicative of binding to the heme iron.

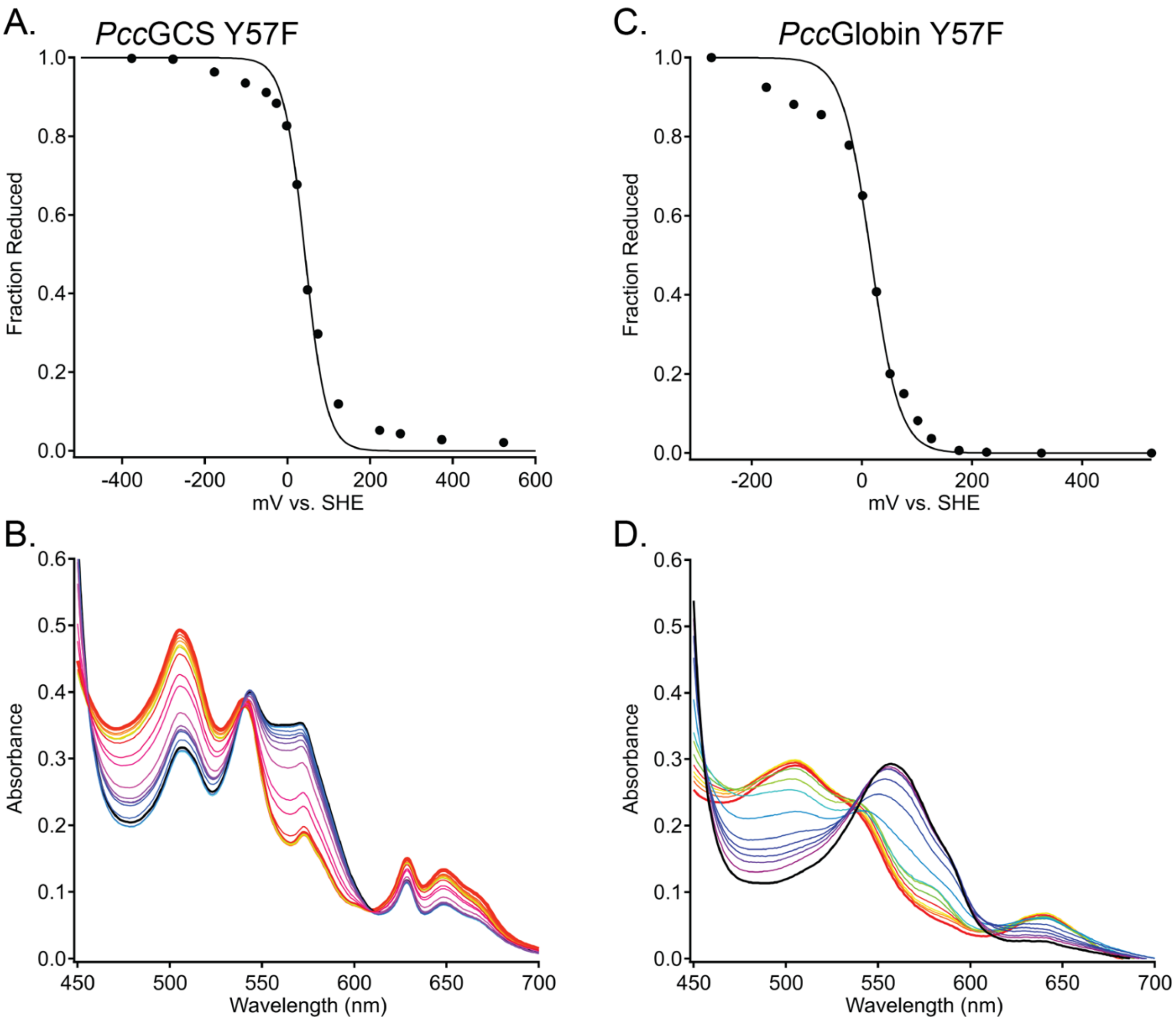

Figure S4: **Electrochemical characterization of *Pcc*GCS Y57F and *Pcc*Globin Y57F variants.** (A) Representative oxidative titration of *Pcc*GCS Y57F in 50 mM HEPES, 50 mM KCl, pH 7.0. Midpoint potentials were determined using the spectral changes at ~570 nm and 640 nm and plotting the fraction of reduced iron against the set potential. The data was fit as previously described to one, reversible redox active component.[6] (B) Absorption spectra of *Pcc*GCS Y57F from oxidative titration in an OTTLE cell. (C) Representative oxidative titration of *Pcc*Globin Y57F in 50 mM HEPES, 50 mM KCl, pH 7.0. Midpoint potentials were determined using the spectral changes at ~570 nm and 640 nm and plotting the fraction of reduced iron against the set potential. The data was fit as previously described to one redox active component. (B) UV-Vis absorption spectra of *Pcc*Globin Y57F from oxidative titration.

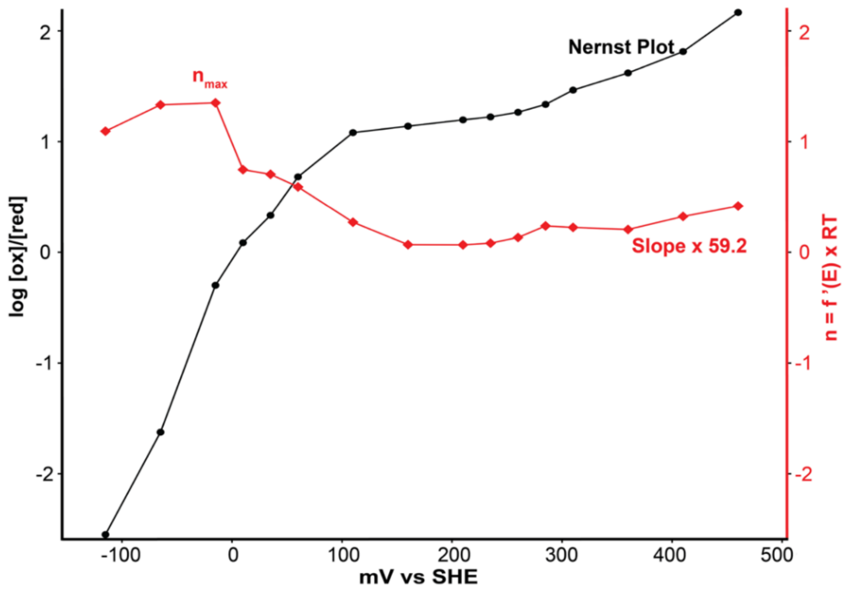

Figure S5: Nernst Plot for PccGCS WT. Representative oxidative titration of PccGCS WT in 50 mM HEPES, 50 mM KCl, pH 7.0. Log[ox]/[red] was determined using the spectral changes at 434 nm by plotting the log of the fraction of the population [oxidized]/[reduced] against the set potential (black). The change in slope multiplied by 59.2 equals the value of n, which is indicative of interaction between subunits, and is interpreted like the Hill value n to indicate cooperativity (red). n_{max} represents the maximum cooperativity value in the electrochemical titration. Five redox titrations were averaged to determine n.

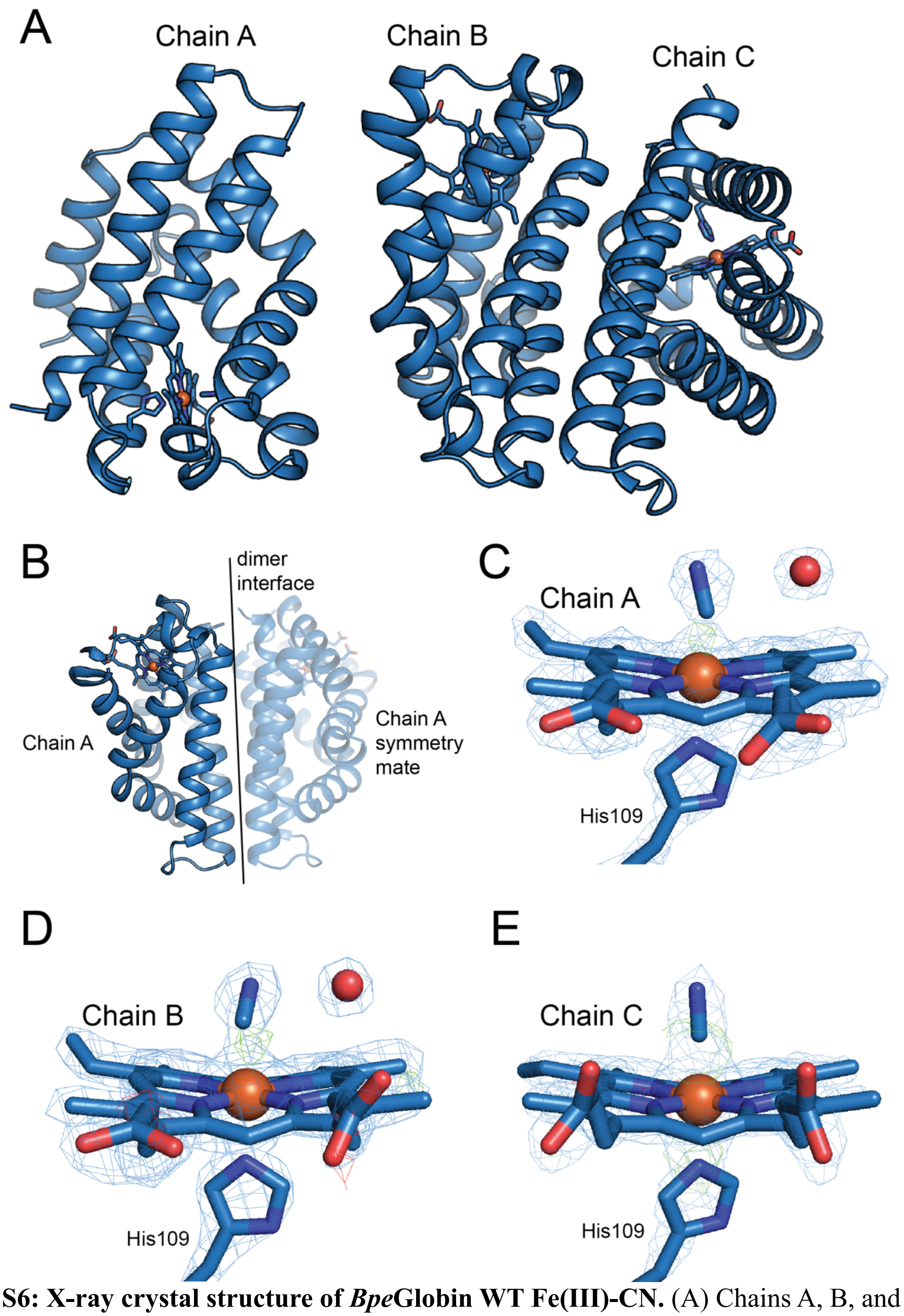

Figure S6: X-ray crystal structure of *Bpe*Globin WT Fe(III)-CN. (A) Chains A, B, and C (B) The monomer of the chain A dimer is situated in the adjacent unit cell. (C-E) Heme from each chain is depicted with Fe(III)-CN, axial His ligand, and ordered distal pocket water (red sphere). $2F_o$-F_c maps (blue mesh) are shown contoured at 1.5σ. F_o-F_c maps (green and red mesh) are shown contoured at 3σ. Fe(III) ions are shown as orange spheres. Green density may be indicative of photoreduction of the Fe(III)-CN complex.

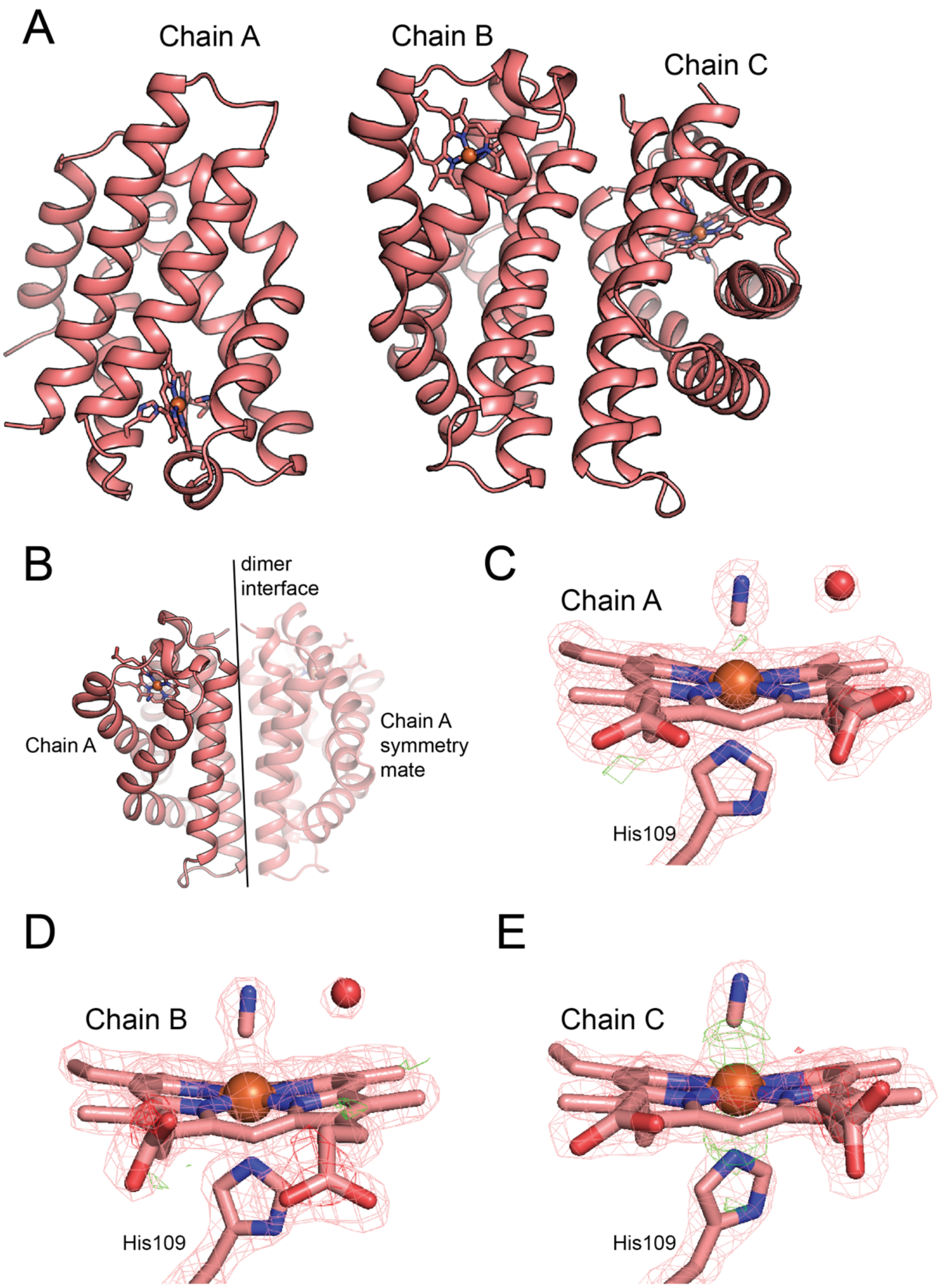

Figure S7: X-ray crystal structure of of *Bpe*Globin S68A Fe(III)-CN. (A) Chains A, B, and C. (B) The monomer of the chain A dimer is situated in the adjacent unit cell. (C-E) Heme from each chain is depicted with Fe(III)-CN, axial His ligand, and ordered distal pocket water (red sphere). 2F_o-F_c maps (pink mesh) are shown contoured at 1.5σ, except the water in Chain B, which is contoured at 0.75σ due to weak density. F_o-F_c maps (green and red mesh) are shown contoured at 3σ. Fe(III) ions are shown as orange spheres. Green density may be indicative of photoreduction of the Fe(III)-CN complex.

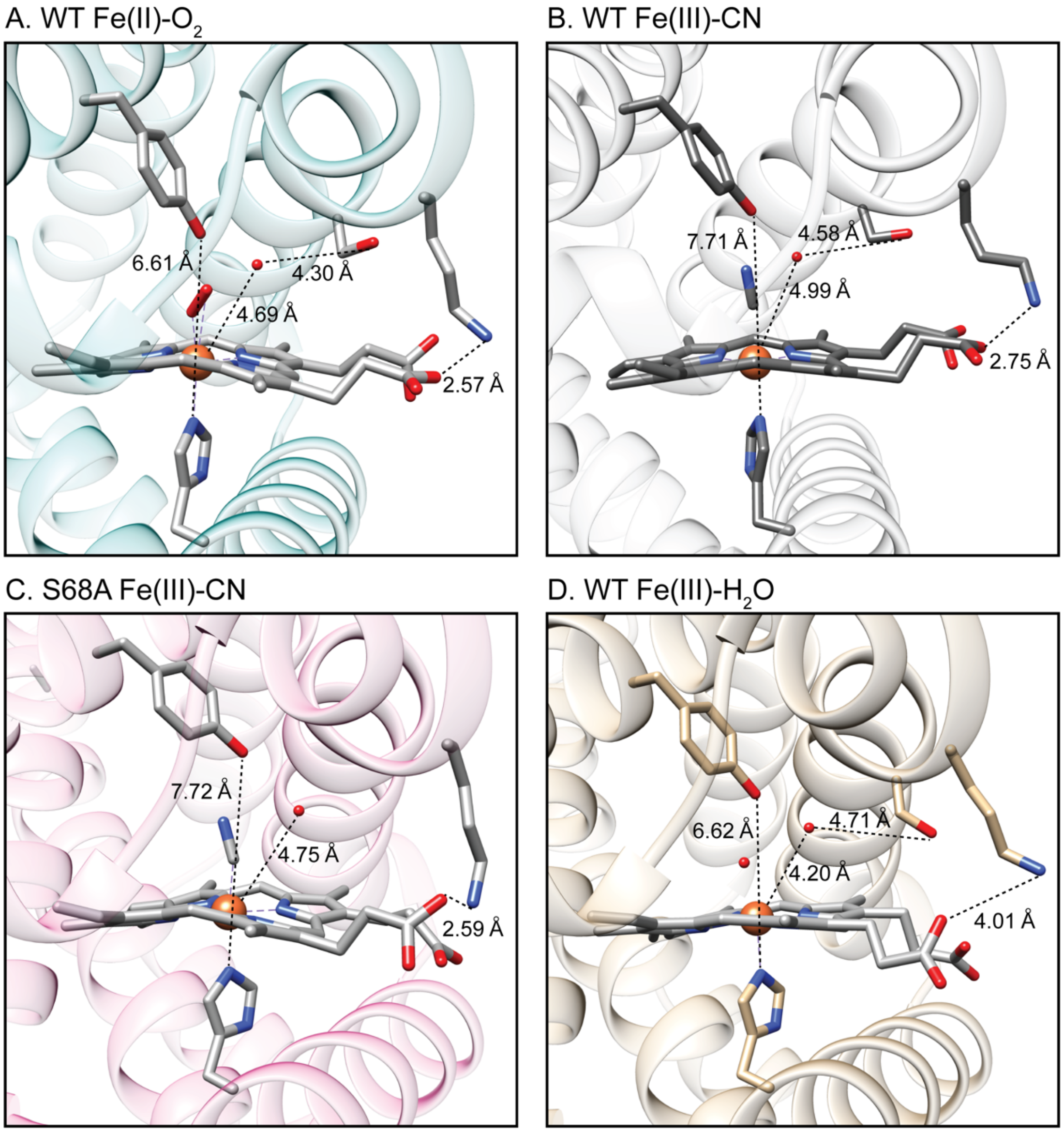

Figure S8: *Bpe*Globin distance comparisons. Distances between distal tyrosine (Y57) and proximal histidine, heme iron and heme pocket water, distal serine and heme pocket water, and heme propionate and hydrogen bonding residue (K64 in *Bpe*Globin, R78 in *Pcc*Globin) for *Bpe*Globin A) WT Fe(II)-O_2 (PDB: 6m9a), B) WT Fe(III)-CN, C) S68A Fe(III)-CN, and D) WT Fe(III)-H_2O (PDB: 6m9a).

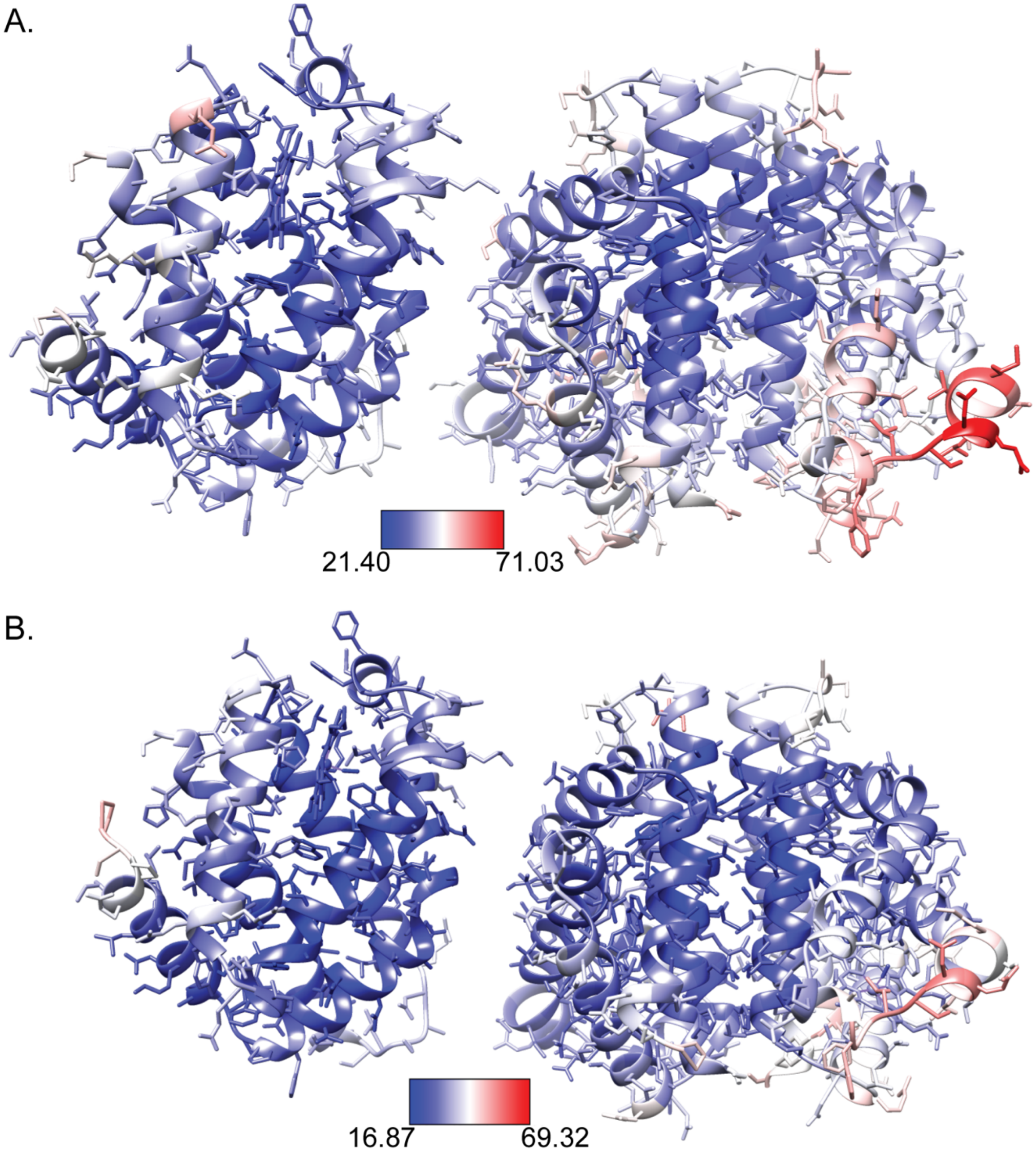

Figure S9: Comparison of B-factors. Residue average B-factors for *Bpe*Globin WT Fe(III)-CN (A.) and S82A Fe(III)-CN (B.) depicted on the respective structures.

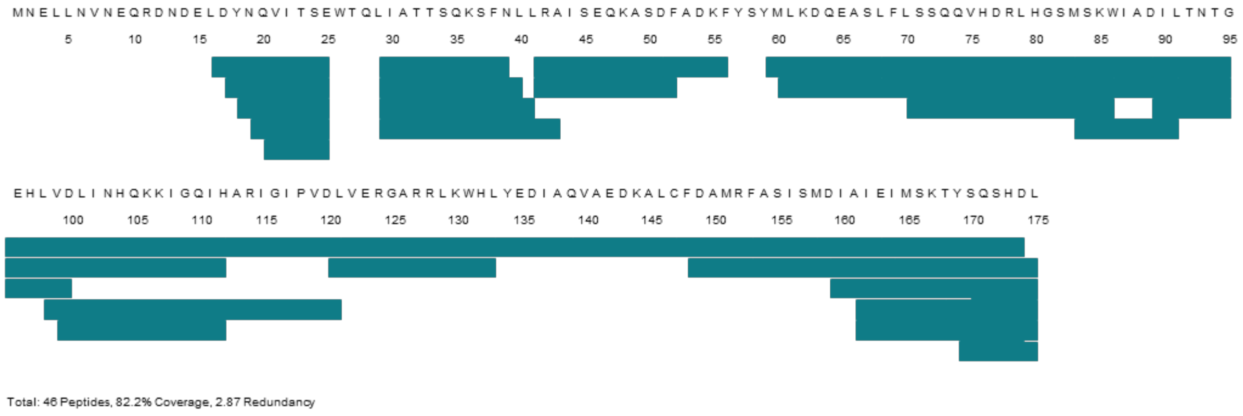

Figure S10: HDX-MS coverage map for *Pcc*Globin WT. Identified peptides are depicted as teal bars.

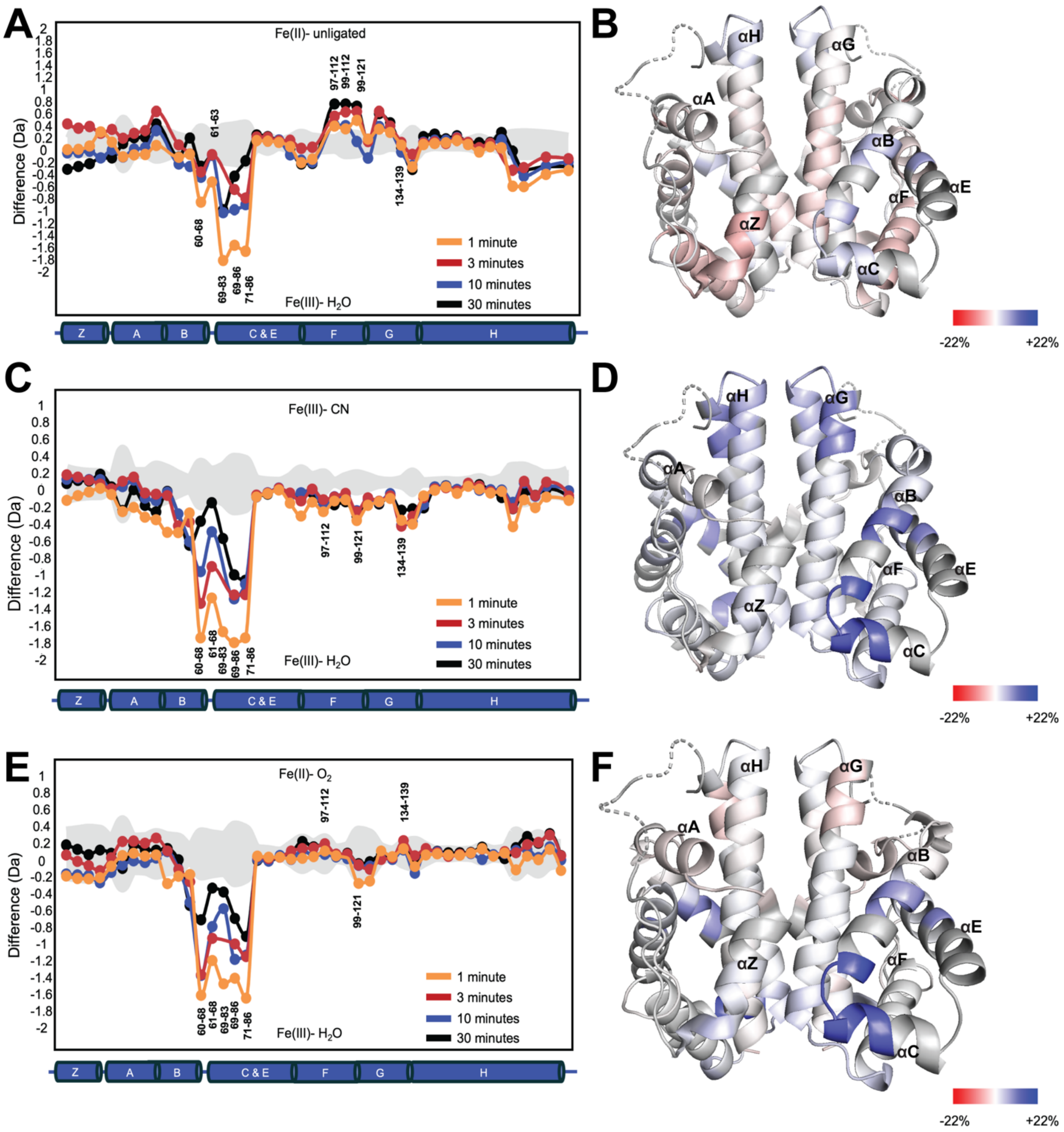

Figure S11: HDX-MS comparison of *Pcc*Globin Fe(II)-O$_2$, Fe(III)-CN and Fe(III)
(A) Deuterium exchange difference plot (for peptide fragment ordered from the N- to C-terminus on the X-axis) for *Pcc*Globin WT in 50 mM Tris, 50 mM NaCl, pH 7.0 buffer in the low activity Fe(II)-unligated vs Fe(III)-H_2O states. Negative differences indicate decreased exchange in the Fe(II)-unligated state. Standard deviations from replicate measurements are in gray. (B) Deuterium exchange differences (t= 3 min) mapped onto the homology model of *Pcc*Globin.(C) Deuterium exchange difference plot (for pepsin fragment peptides from the N- to C- terminus (X-axis) for *Pcc*Globin WT in the Fe(III)-CN vs Fe(III)-H_2O states. Negative differences indicate decreased exchange (blue boxes) in the Fe(III)-CN state. (D) Deuterium exchange differences (t= 3 min) mapped onto the homology model of *Pcc*Globin.

(E) Deuterium exchange difference plot (for pepsin fragment peptides from the N- to C- terminus (X-axis) for *Pcc*Globin WT in the Fe(II)-O_2 vs Fe(III)-H_2O states. Negative differences indicate decreased exchange (blue boxes) in the Fe(II)-O_2 state. (F) Deuterium exchange differences (t= 3 min) mapped onto the homology model of *Pcc*Globin.

Table S1. Results from fitting equilibrium O_2 titrations to a two-site model. Values are the average of at least three titrations.
f(x) = ((K1*x)+(2*K1*K2*(x^2)))/((1+ K1*x)+(K1*K2*(x^2)))

**Protein**	**K1 ± Std. Dev**	**K2 ± Std. Dev**
*Pcc*GCS WT	1.04 ± 0.59	0.018 ± 0.011
*Pcc*Globin WT	0.94 ± 0.32	0.013 ± 0.004

Table S2: Spectroelectrochemical results from PccGCS WT and tyrosine variants. Values are reported as the average of 2-3 experiments ± S.D.

**Protein**	**Midpoint 1 (mV vs SHE)**	**Midpoint 2 (mV vs SHE)**	**n max**
hhMb[7]	37	-	0.95
swMb[7]	47	-	0.9
*Pcc*GCS WT[6]	−7 ± 21	246 ± 64	1.1 +/- 0.3
*Pcc*GCS Y57F	42 ± 3	-	ND
*Pcc*Globin Y57F	19 ± 3	-	1.2 +/- 0.2

Table S3: Data collection and refinement statistics for x-ray structures of BpeGlobin WT and BpeGlobin S68A

	BpeGlobin WT	BpeGlobin S68A
**Wavelength**	0.97872 Å	0.9793
**Resolution range**	39.97 - 2.08 (2.154 - 2.08)	46.67 - 1.85 (1.916 - 1.85)
**Space group**	C 1 2 1	C 1 2 1
**Unit cell**	145.136, 49.237, 67.828, 90, 110.479, 90	144.958, 49.702, 67.994, 90, 110.556, 90
**Total reflections**	187461	899144
**Unique reflections**	27172 (2692)	38802 (3885)
**Multiplicity**	3.7 (2.9)	3.4 (3.4)
**Completeness (%)**	99.48 (99.41)	99.48 (99.64)
**Mean I/sigma(I)**	22.2 (2.3)	21.1 (2.0)
**Wilson B-factor**	30.38	26.73
**R-pim**	0.056 (0.266)	0.026 (0.210)
**CC1/2**	0.913 (0.84)	0.992 (0.886)
**Reflections used in refinement**	27134 (2692)	38795 (3885)
**Reflections used for R-free**	1326 (125)	1896 (184)
**R-work**	0.2127 (0.2632)	0.2112 (0.2665)
**R-free**	0.2601 (0.3067)	0.2423 (0.2804)
**Number of non-hydrogen atoms**	3885	3898
**macromolecules**	3630	3647
**ligands**	143	141
**solvent**	112	110
**Protein residues**	464	467
**RMS(bonds)**	0.016	0.016
**RMS(angles)**	1.65	1.62
**Ramachandran favored (%)**	98.47	98.48
**Ramachandran allowed (%)**	1.53	1.52
**Ramachandran outliers (%)**	0.00	0.00
**Rotamer outliers (%)**	0.53	0.27
**Clashscore**	2.70	1.48
**Average B-factor**	36.08	29.44
**macromolecules**	36.13	29.47
**ligands**	35.29	28.09
**solvent**	35.56	30.27

*Values in parentheses are for highest resolution shell.

Table S4: Fe-CN bond parameters for GCS proteins. Comparison of key bond length and angles for Fe(III)-CN sensor globins.

	Chain	Fe-His Bond (Å)	Fe-C-N Bond (Å)	Fe-C-N Angle (degrees)
***Bpe*Globin WT (this work; 6DSE)**	A	2.13	2.11	160.5
	B	2.1	2.34	124.4
	C	2.06	2.04	175.8
***Bpe*Globin S82A (this work; 6DSF)**	A	2.05	1.88	164.6
	B	2.12	1.89	154.5
	C	2.11	1.88	171.9
**HemAT*bs* (1OR4)**	A	2.01	1.62	173.34
	B	2	1.49	164.1
***Af*GcHK (5OHE)**	A	2.25	2.02	162.1
	B	2.09	2.08	145
	C	2.01	2.31	145.7
	D	*water		
	E	2.17	1.94	170.9
	F	*water		
	G	2.16	2.1	137.9
	H	2.14	2.13	153.8
**swMb (1EBC)**	A	2.02	2.02	166.13

Table S5: NSD Heme Deformation Data of GCS Proteins. NSD values were calculated using the Kingsbury, C.J. et al program. [8] Total structural decomposition methods are reported below.

**Protein**	**Ligation State**	**pdb code**	**Chain**	**Δoop (all over)**	**δ oop**	**B2u (saddle)**	**B1u (ruffle)**	**A2u (dome)**	**Eg(x) (wave)**	**Eg(y) (wave)**	**A1u (propeller)**
*Bpe*Globin	Fe(II)-O_2	6m9a	A	1.21	0	0.27	-1.12	0.25	-0.15	-0.05	-0.02
*Bpe*Globin	Fe(II)-O_2/H_2O	6m9a	B	1.07	0	0.13	1.02	-0.17	-0.17	-0.03	0.01
*Bpe*Globin	Fe(III)-H_2O	6m9a	C	0.89	0	0.18	-0.85	-0.13	-0.08	0.05	0
*Bpe*Globin	Fe(III)-CN	this work	A	1.12	0	0.07	-1.11	-0.03	0	0	0
*Bpe*Globin	Fe(III)-CN	this work	B	1.24	0	0.12	1.22	0.02	-0.01	0	0
*Bpe*Globin	Fe(III)-CN	this work	C	1.27	0	-0.01	1.25	-0.15	-0.01	0.01	0
*Bpe*Globin	Fe(III)-CN	this work	A	1.22	0	-0.11	1.21	0.03	0.01	0	0
*Bpe*Globin	Fe(III)-CN	this work	B	1.39	0	0.12	1.37	-0.06	-0.01	0	0
*Bpe*Globin	Fe(III)-CN	this work	C	1.35	0	0.02	-1.34	0.01	0	0	0
HemAT*bs*	Fe(III)-CN	1or4	A	0.39	0	0.22	0.13	0.05	-0.07	-0.01	-0.02
HemAT*bs*	Fe(III)-CN	1or4	B	0.52	0	0.36	-0.11	-0.12	-0.12	-0.1	-0.08
HemAT*bs*	Fe(III)-H_2O	1or6	A	0.61	0	-0.25	0.48	0.21	0.08	0.01	0.09
HemAT*bs*	Fe(III)-H_2O	1or6	B	0.58	0	-0.05	0.45	-0.36	0.01	-0.02	-0.03
*Ec*DosC	Fe(II)	4zvb	A	0.81	0	-0.16	-0.58	0.49	-0.09	0.03	0.01
*Ec*DosC	Fe(II)	4zvb	B	1.01	0	-0.01	-0.76	0.63	-0.03	0.01	0.01
*Ec*DosC	Fe(II)	4zvb	C	0.99	0	-0.11	0.76	-0.57	0.02	-0.11	0.01
*Ec*DosC	Fe(II)	4zvb	D	0.99	0	-0.05	-0.75	-0.59	-0.08	-0.01	0.01
*Ec*DosC	Fe(III)	4zva	A	0.9	0	0.02	0.68	-0.53	0.11	0.1	0.01
*Ec*DosC	Fe(III)	4zva	B	0.99	0	-0.05	0.72	0.62	-0.13	0.04	0.01
*Af*GcHK	Fe(III)	5ohf	A	1.02	0	0.15	-0.99	-0.15	0.04	0.02	0.01

*Af*GcHK	Fe(III)	5ohf	B	0.89	0	-0.2	-0.85	0.12	-0.04	0.01	0.01
*Af*GcHK	Fe(III)	5ohf	C	0.78	0	-0.17	-0.75	0.11	-0.02	0.01	0
*Af*GcHK	Fe(III)	5ohf	D	0.9	0	-0.16	-0.87	0.12	-0.03	0.01	0.01
*Af*GcHK	Fe(III)	5ohf	E	0.95	0	-0.28	0.88	-0.18	0	-0.01	0.02
*Af*GcHK	Fe(III)	5ohf	F	0.97	0	-0.22	-0.92	0.18	0.03	-0.01	0.01
*Af*GcHK	Fe(III)	5ohf	G1	0.88	0	0.13	0.86	0.04	-0.01	0.02	0
*Af*GcHK	Fe(III)	5ohf	G2	0.6	0	-0.08	0.59	-0.03	0.01	-0.02	0
*Af*GcHK	Fe(III)	5ohf	H	0.98	0	-0.27	-0.91	0.19	-0.05	0	0.01
*Af*GcHK	Fe(III)-CN	5ohe	A	0.86	0	-0.19	0.83	-0.05	-0.01	-0.04	0.02
*Af*GcHK	Fe(III)-CN	5ohe	B	0.91	0	-0.23	-0.87	0.1	0.04	0.02	0.02
*Af*GcHK	Fe(III)-CN	5ohe	C	0.83	0	-0.18	-0.79	0.13	-0.03	0	0
*Af*GcHK	Fe(III)-CN	5ohe	D	0.86	0	-0.16	-0.82	0.13	0.02	-0.03	0.02
*Af*GcHK	Fe(III)-CN	5ohe	E	0.87	0	0.32	-0.77	-0.2	-0.03	-0.03	0
*Af*GcHK	Fe(III)-CN	5ohe	F	0.95	0	0.21	0.9	0.17	0	-0.06	0.01
*Af*GcHK	Fe(III)-CN	5ohe	G	0.67	0	-0.17	-0.62	0.13	0.02	-0.02	0.02
*Af*GcHK	Fe(III)-CN	5ohe	H	0.98	0	-0.27	-0.91	0.17	-0.01	0.01	-0.01

Table S6: Relative Deuterium Uptake. Comparison of relative deuterium uptake for peptide 60-68 in the four ligation states at 0, 1, 3, 10, and 30 minutes.

State	Exposure (min)	Uptake (RFU)	StDev
Fe(III)-H_2O	0	0	0.0279
Fe(III)-H_2O	1	3.1011	0.2499
Fe(III)-H_2O	3	3.2059	0.0956
Fe(III)-H_2O	10	3.6312	0.107
Fe(III)-H_2O	30	3.7513	0.1232
Fe(III)-CN	0	0	0.039
Fe(III)-CN	1	1.2914	0.0322
Fe(III)-CN	3	1.8	0.0636
Fe(III)-CN	10	2.6014	0.0785
Fe(III)-CN	30	3.3129	0.1061
Fe(II)-O_2	0	0	0.0362
Fe(II)-O_2	1	1.4164	0.0595
Fe(II)-O_2	3	1.6383	0.0407
Fe(II)-O_2	10	2.166	0.1034
Fe(II)-O_2	30	2.9662	0.0377
Fe(II)-unligated	0	0	0.0544
Fe(II)-unligated	1	2.145	0.0667
Fe(II)-unligated	3	2.523	0.3738
Fe(II)-unligated	10	3.0869	0.1188
Fe(II)-unligated	30	3.262	0.2495

References

(1) Rivera, S.; Young, P. G.; Hoffer, E. D.; Vansuch, G. E.; Metzler, C. L.; Dunham, C. M.; Weinert, E. E. Structural Insights into Oxygen-Dependent Signal Transduction within Globin Coupled Sensors. *Inorg Chem* **2018**, *57* (22), 14386–14395. https://doi.org/10.1021/acs.inorgchem.8b02584.

(2) Tarnawski, M.; Barends, T. R. M.; Schlichting, I. Structural Analysis of an Oxygen-Regulated Diguanylate Cyclase. *Acta Crystallogr D Biol Crystallogr* **2015**, *71*, 2158–2177. https://doi.org/10.1107/S139900471501545X.

(3) Zhang, W.; Phillips, G. N. Structure of the Oxygen Sensor in Bacillus Subtilis: Signal Transduction of Chemotaxis by Control of Symmetry. *Structure* **2003**, *11* (9), 1097–1110. https://doi.org/10.1016/S0969-2126(03)00169-2.

(4) Skalova, T.; Lengalova, A.; Dohnalek, J.; Harlos, K.; Mihalcin, P.; Kolenko, P.; Stranava, M.; Blaha, J.; Shimizu, T.; Martínková, M. Disruption of the Dimerization Interface of the Sensing Domain in the Dimeric Heme-Based Oxygen Sensor AfGcHK Abolishes Bacterial Signal Transduction. *Journal of Biological Chemistry* **2020**, *295* (6), 1587–1597. https://doi.org/10.1074/jbc.RA119.011574.

(5) Stranava, M.; Man, X. P.; Skálova, X. T.; Kolenko, X. P.; Blaha, X. J.; Fojtikova, X. V.; Martínek, X. V.; Dohnálek, X. J.; Lengalova, A.; Rosulek, X. M.; Shimizu, T.; Martínková, X. M. Coordination and Redox State-Dependent Structural Changes of the Heme-Based Oxygen Sensor AfGcHK Associated with Intraprotein Signal Transduction. *Journal of Biological Chemistry* **2017**, *292* (51), 20921–20935. https://doi.org/10.1074/jbc.M117.817023.

(6) Patterson, D. C.; Ruiz, M. P.; Yoon, H.; Walker, J. A.; Armache, J. P.; Yennawar, N. H.; Weinert, E. E. Differential Ligand-Selective Control of Opposing Enzymatic Activities within a Bifunctional c-Di-GMP Enzyme. *Proc Natl Acad Sci U S A* **2021**, *118* (36). https://doi.org/10.1073/pnas.2100657118.

(7) Faulkner, K. M.; Bonaventura, C.; Crumbliss, A. L. A Spectroelectrochemical Method for Differentiation of Steric and Electronic Effects in Hemoglobins and Myoglobins. *Journal of Biological Chemistry* **1995**, *270* (23), 13604–13612. https://doi.org/10.1074/jbc.270.23.13604.

(8) Kingsbury, C. J.; Senge, M. O. The Shape of Porphyrins. *Coord Chem Rev* **2021**, *431*, 213760. https://doi.org/10.1016/j.ccr.2020.213760.
